# HACD2 Promotes Pancreatic Cancer Progression by Enhancing PKM2 Dissociation From PRKN in a Dehydratase‐Independent Manner

**DOI:** 10.1002/advs.202407942

**Published:** 2025-01-21

**Authors:** Xuanning Chu, Jinyu Zhao, Yuting Shen, Qi Feng, Changlin Zhou, Lingman Ma, Yiran Zhou

**Affiliations:** ^1^ School of Life Science and Technology China Pharmaceutical University Nanjing Jiangsu 211198 China; ^2^ Department of General Surgery Pancreatic Disease Center Ruijin Hospital Shanghai Jiaotong University School of Medicine 197 Ruijin Er Road Shanghai 200025 China; ^3^ Research Institute of Pancreatic Disease Shanghai Jiao Tong University School of Medicine Shanghai 200025 China

**Keywords:** HACD2, pancreatic cancer, PKM2, PRKN, ubiquitination

## Abstract

3‐Hydroxyacyl‐CoA dehydratase 2 (HACD2), an obesity‐related gene involved in the elongation of long‐chain fatty acids, is highly expressed in pancreatic cancer (PC) and is associated with patient prognosis. Interestingly, the study reveals that HACD2 mediated the proliferation of PC cells in a dehydratase‐independent manner, affecting the downstream glycolytic pathway. Mechanistically, HACD2 promotes PC cells proliferation by binding to E3 ubiquitin‐protein ligase parkin (PRKN) and enhancing pyruvate kinase PKM (PKM2) dissociation from PRKN, resulting in reduced ubiquitination of PKM2 and increased dimerization of PKM2, which subsequently promote c‐Myc expression and tumor growth. Moreover, HACD2 overexpression‐induced PC growth is mitigated by knockdown of PKM2 or overexpression of PRKN. Furthermore, the weight loss drug orlistat, which potentially binds to HACD2, disrupted the interaction between HACD2 and PRKN and further increased the ubiquitination of PKM2. Therefore, this study elucidates the mechanism by which the obesity‐related gene HACD2 regulates PC cells proliferation through a noncanonical signaling pathway, which may provide a potential new target and strategy for the individualized clinical treatment of PC.

## Graphical abstract

3‐Hydroxyacyl‐CoA dehydratase 2 (HACD2) promotes pancreatic cancer progression by binding to PRKN and promoting dissociating PKM2 from PRKN in a dehydratase‐independent manner, resulting in a decrease in PKM2 ubiquitination and an increase in PKM2 dimerization, followed by the promotion of c‐Myc expression and tumor growth. Moreover, orlistat achieves antitumor effect by directly binding to HACD2 and increasing the binding of PKM2 to PRKN.

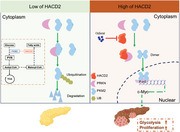

## 1 Introduction

Pancreatic cancer (PC) is extremely malignant, and the overall 5‐year survival rate is less than 10%, this disease has surpassed breast cancer as the third leading cause of cancer‐related death, according to the latest data from the American Cancer Society.^[^ ^1^, ^2^, ^3^, ^4^ ^]^ Obesity is a global pandemic that has been associated with the development of PC.^[^ ^5^, ^6^ ^]^ An increasing number of studies have shown that obesity‐related genes, such as fatty acid synthase and G protein‐coupled receptors, play important roles in regulating the occurrence and development of PC.^[^ ^7^, ^8^ ^]^

3‐Hydroxyacyl‐CoA dehydratase 2 (HACD2) is a key dehydratase in the endoplasmic reticulum that regulates the elongation of long‐chain fatty acids and catalyzes the dehydration of 3‐hydroxyacyl‐CoA intermediates to trans‐2, 3‐enoyl‐CoA;^[^ ^9^, ^10^ ^]^ this molecule is widely expressed in human tissues and as on saturated, monounsaturated, and polyunsaturated 3‐hydroxyacyl‐CoA of long‐to very long‐chain fatty acids (VLCFAs).^[^ ^9^, ^10^ ^]^ Studies have shown that HACD2 is highly expressed in obese patients and that liver‐specific HACD2 deletion protects mice from diet‐induced obesity.^[^ ^11^, ^12^, ^13^, ^14^ ^]^ In addition, HACD2 is highly expressed in various tumors and is associated with patient prognosis.^[^ ^15^, ^16^ ^]^ Recent studies have shown that the deletion of HACD2 causes vascular retardation, growth inhibition, chemotherapeutic drug resistance, and embryonic lethality in mouse embryos.^[^ ^17^ ^]^ Therefore, further elucidation of the potential molecular mechanism of HACD2 in the progression of PC will contribute to the development of biomarker‐driven therapeutic targets for individual PC patients.

Cancer cells achieve rapid proliferation and immune escape through metabolic reprogramming, and the reprogramming of lipid metabolism and glucose metabolism is the most common feature of PC cells.^[^ ^18^, ^19^ ^]^ Given the importance of lipid synthesis in tumor proliferation, HACD2 may promote the occurrence and development of PC by increasing fatty acid synthesis. Interestingly, some enzymes in metabolic pathways can perform their biological functions without their own enzymatic activity. An increasing number of studies have shown that tumor cells can not only regulate the classic functions of metabolic enzymes to meet their needs for rapid proliferation but also regulate various complex cellular activities and the occurrence and development of diseases through the nonclassic/nonmetabolic functions of enzymes,^[^ ^20^, ^21^, ^22^ ^]^ indicating that HACD2 can promote the proliferation of PC cells through its nonenzymatic activity by binding other proteins.

In this study, we reported that high HACD2 expression in PC is positively correlated with tumor growth. HACD2 promotes PC proliferation by blocking the ubiquitination of pyruvate kinase PKM (PKM2) via binding to its E3 ubiquitin‐protein ligase parkin (PRKN). In addition, our study revealed that orlistat, which has a high affinity for HACD2, blocked the binding of HACD2 to PRKN and inhibited the proliferation of PC cells. Together, these data identify the HACD2/PRKN/PKM2 signaling axis and provide a potential new target for the individualized clinical treatment of PC.

## 2 Results

### 2.1 HACD2 Expression is Positively Correlated with PC Progression

**Figure 1:**
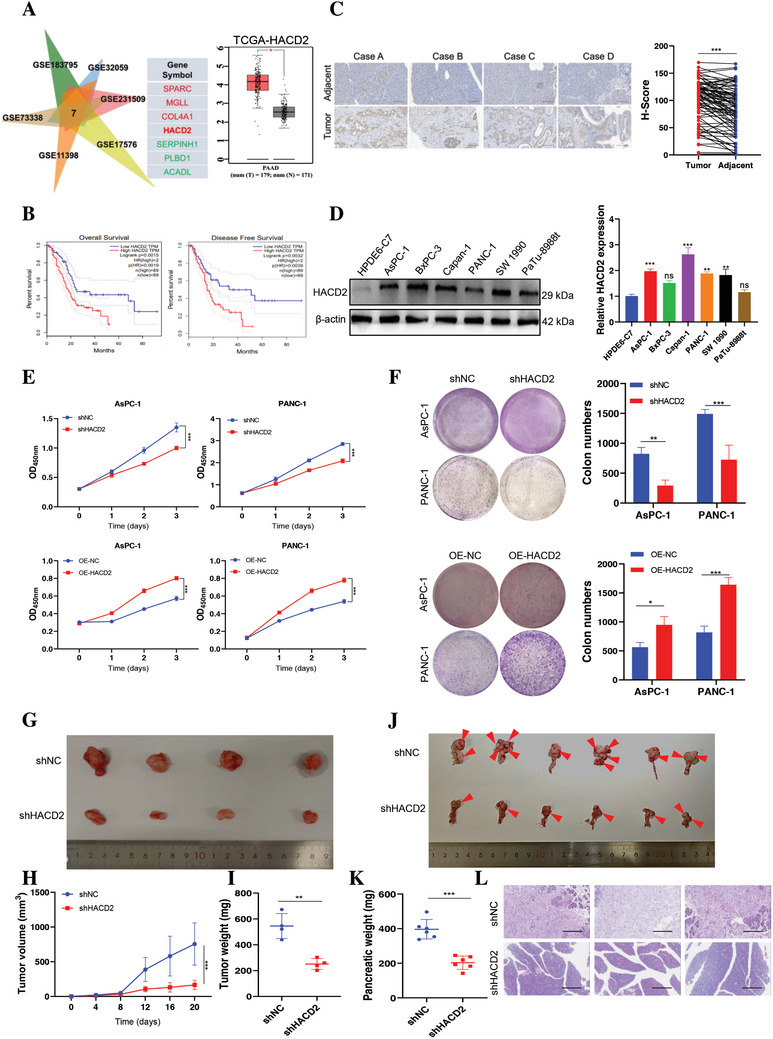
HACD2 expression is positively correlated with PC progression. A) Scheme for the identification of target genes involved in PC patients and patients with obesity. DEGs with *p* < 0.05 in each dataset are shown in the Venn diagram (left). HACD2 expression in pancreatic cancer and normal tissues was analyzed with the GEPIA database (right) (https://www.ncbi.nlm.nih.gov/). B) Kaplan–Meier analysis via GEPIA revealed that HACD2 expression levels are correlated with poor overall survival and progression‐free survival in pancreatic cancer patients (http://gepia2.cancer‐pku.cn). C) Representative images and quantification of immunohistochemistry staining of HACD2 in paired paracarcinoma and primary tumors from tissue chip, *n* = 83. Data were presented as means ± SD. *p < 0.05 according to paired two‐tailed Student's *t*‐tests. Scale bar, 100 µm. D) HACD2 expression in different pancreatic cancer cell lines was verified by RT‒qPCR (right, *n* = 4) and western blotting (left). Data were presented as means ± SD. ns., not significant; **p < 0.01, ***p < 0.001, according to one‐way ANOVA. E,F) CCK‐8 (*n* = 4) and colony formation (*n* = 3) assays were used to assess cell viability and proliferation after HACD2 knockdown or overexpression. Data were presented as means ± SD. *p < 0.05, **p < 0.01, ***p < 0.001 according to two‐way ANOVA and unpaired two‐tailed Student's *t*‐test. PANC‐1‐related stable cells (control, shHACD2) were injected into the right flanks of null mice. G–I) Tumor volume was measured every 4 days. Images of tumor growth curves and weights were obtained after dissection on day 20. Data were presented as means ± SD, *n* = 4. **p < 0.01, ***p < 0.001 according to two‐way ANOVA and unpaired two‐tailed Student's *t*‐test; PANC‐1‐related stable cells (control and shHACD2) were injected into the pancreata of nude mice. J,K) Pancreatic images and weights were obtained after dissection at week 8. The red arrow indicates the tumor tissue in the pancreas, *n* = 6. Data were presented as means ± SD. ***p < 0.001 according to unpaired two‐tailed Student's *t*‐test. L) H&E staining of orthotopically transplanted tumors, *n* = 3, scale bar, 200 µm.

To explore potential risk factors for PC, we analyzed the differential expression of genes related to PC and obesity in the Gene Expression Omnibus (GEO) database (PC: GSE11398, GSE73338, and GSE183795; obese: GSE32059, GSE231509, and GSE17576). Among the 7 differentially expressed genes (DEGs) identified, 4 were upregulated and 3 were downregulated (**Figure** **1**A). Interestingly, only HACD2 was significantly highly expressed in PC and was found to be correlated with patient prognosis (Figure 1A,B; and Figure S1A,B, Supporting Information). Similarly, the findings from the tissue chip indicated that HACD2 was highly expressed in PC tumors, particularly in stage II patients, and those with elevated HACD2 expression were more likely to have a poor prognosis (Figure 1C; and Table S1, Supporting information). Western blotting and quantitative real‐time PC (RT‐qPCR) demonstrated elevated expression levels of HACD2 in PC cells compared with normal pancreatic epithelial cells (Figure 1D). Notably, higher levels of HACD2 were also observed in other human tumors, but these groups did not significantly differ in terms of HACD2 expression or survival rates (Figure S1C,D, Supporting Information). Analysis via the GEPIA public database for other isoforms in the HACD family indicated that although their expression levels were increased in PC, the expression did not significantly affect overall patient survival rates (Figure S1E,F, Supporting Information). These findings confirmed that HACD2 may be a biomarker for assessing the progression of PC.

To further elucidate the biological function of HACD2 in PC, we ectopically expressed and silenced HACD2 in the PANC‐1 and AsPC‐1 cell lines (Figure S2A–D, Supporting Information) and subsequently examined its effect on PC cell proliferation through Cell Counting Kit‐8 (CCK‐8) and plate cloning assays. The results confirmed that HACD2 knockdown inhibited the proliferation of PC cells, whereas HACD2 overexpression had the opposite effect (Figure 1E,F). These findings verified that HACD2 promoted the proliferation of PC cells in vitro. Moreover, HACD2 knockdown reduced the migration and apoptosis of PC cells, although the impact on apoptosis was not substantial (Figure S2E,F, Supporting Information). To investigate the effect of HACD2 on the progression of PC in vivo, we generated a subcutaneous xenograft model of HACD2 knockdown PANC‐1 cells. Consistent with the results of the in vitro experiments, the volume and weight of the subcutaneously transplanted tumors significantly decreased after HACD2 knockdown (Figure 1G–I). This result was further verified in orthotopic tumors, where low expression of HACD2 inhibited orthotopic tumor proliferation (Figure 1J,K), and H&E staining of orthotopic tumors demonstrated that HACD2 knockdown alleviated the lesion effect on PC (Figure 1L). Taken together, these findings indicated that HACD2 plays a key role in the proliferation of PC cells and can be used as a biomarker for PC progression.

### 2.2 HACD2 Promotes PC Cells Proliferation in a Nonenzymatic Manner

**Figure 2:**
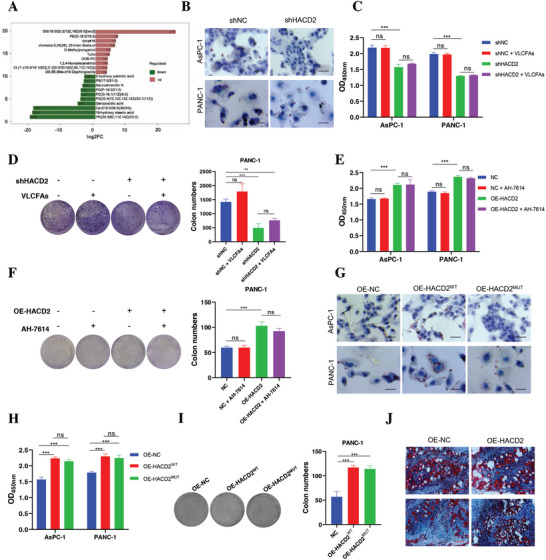
HACD2 promotes PC cells proliferation in a nonenzymatic manner. A) Enrichment analysis of the significantly altered metabolites from PANC‐1 cells (vector control or HACD2 knockdown). *p* < 0.05, unpaired two‐tailed Student's *t*‐test. Red indicates an increase, and green indicates a decrease, *n* = 4. B) Representative images of Oil Red O staining of AsPC‐1 and PANC‐1 cells (vector control or HACD2 knockdown). The cells were analyzed 48 h after adherence. Scale bars, 20 µm. C,D) Quantification of the proliferation of AsPC‐1 and PANC‐1 cells treated for 48 h with 2 µm VLCFAs or vehicle via CCK‐8 (*n* = 4) and colony formation (*n* = 3) assays. Data were presented as means ± SD. ns., not significant; **p < 0.01, ***p < 0.001 according to one‐way ANAVO. E,F) Quantification of the proliferation of AsPC‐1 and PANC‐1 cells treated for 24 h with 50 nm AH‐7614 or vehicle via CCK‐8 (*n* = 4) and in colony formation (*n* = 3) assays. Data were presented as means ± SD. ns., not significant; ***p < 0.001 according to one‐way ANOVA. G) Representative images of Oil Red O staining in AsPC‐1 and PANC‐1 cells transduced with HACD2^WT^, HACD2^MUT^, or a vector control. H,I) The cells were analyzed 48 h after adherence. Scale bar, 20 µm. CCK‐8 (*n* = 4) and in colony formation (*n* = 3) assays were used to quantify the proliferation of AsPC‐1 and PANC‐1 cells transduced with HACD2^WT^, HACD2^MUT^, or a vector control. Data were presented as means ± SD. ns., not significant; ***p < 0.001 according to one‐way ANOVA. J) Representative images of Oil Red O staining of tumor tissue from PANC‐1 cells transduced with control vector or HACD2^WT^ stable cells. Scale bar, 100 µm.

An enrichment analysis of DEGs between PC tissues and normal tissues in the GEO database (GSE11979, GSE62452, GSE28735, GSE211398, GSE183795) revealed that lipid synthesis was crucial for PC progression (Figure S3A,B, Supporting Information). As a dehydratase in the fatty acid synthesis pathway, HACD2 plays an important role in the regulation of fatty acid elongation, and its deletion can decrease 87.4% of VLCFAs in PANC‐1 cells (**Figure** **2**A,B; and Figure S3C,D, Supporting Information). To determine whether the inhibition of PC cells proliferation by HACD2 knockdown was due to the inhibition of fatty acid synthesis, we assessed treatment with VLCFAs (C22:0) and found that it did not rescue the inhibitory effect of HACD2 knockdown on PC cells proliferation (Figure 2C,D). Similarly, treatment with a long‐chain fatty acid receptor inhibitor (GPR120, AH‐7614) also failed to block the proliferation of HACD2‐overexpression PC cells (Figure 2E,F), indicating that the dehydratase activity of HACD2 is not essential for PC cells proliferation.

The tyrosine at position 176 and glutamicacid at position 183 are the enzymatically active sites of HACD2.^[^ ^23^ ^]^ We investigated the effect of HACD2 on PC cells proliferation independent of dehydratase activity by mutating the two enzyme active sites simultaneously. Mutation of HACD2 effectively blocked fatty acid production (Figure 2G; and Figure S3E, Supporting Information) but did not disrupt the proliferation of PC cells in response to HACD2 (Figure 2H,I). Oil Red O staining further revealed that lipid levels in the tumor tissues of the HACD2‐overexpressing tumor‐bearing mice were not significantly different from those in the control group (Figure 2J), indicating that the excessive PC cells proliferation caused by HACD2 overexpression is not dependent on lipid accumulation. Collectively, these results demonstrated that HACD2 promoted the proliferation of PC cells in a nonenzymatic manner.

### 2.3 PKM2‐Mediated Glycolysis is Inhibited after HACD2 Knockdown

**Figure 3:**
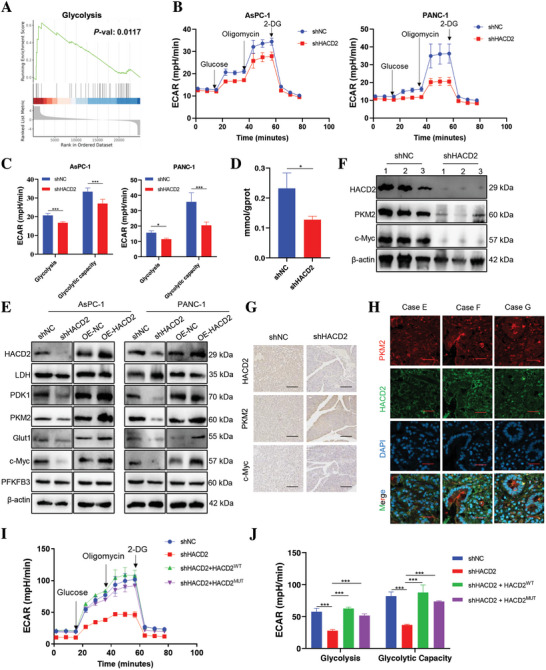
PKM2‐mediated glycolysis is inhibited after HACD2 knockdown. A) Gene set enrichment analysis showing the enrichment of HACD2 target genes. PANC‐1 cells were transfected with scrambled or HACD2 overexpression plasmids, *n* = 4. B) The ECAR was measured in cells transfected with shHACD2 or shNC via an XF Extracellular Flux Analyzer, *n* = 8. C) Statistical analysis of the effects of HACD2 knockdown on glycolytic activity, *n* = 8. Data were presented as means ± SD. ***p < 0.001 according to unpaired two‐tailed Student's *t*‐test. D) Relative lactate release in HACD2‐knockdown tumor tissue from PANC‐1 tumor‐bearing mice, *n* = 3. Data were presented as means ± SD. *p < 0.05, according to unpaired two‐tailed Student's *t*‐test. E) The protein expression levels of glycolysis‐related genes were assessed by western blotting in AsPC‐1 and PANC‐1 cells after HACD2 knockdown or overexpression. F) Western blots were used to assess the expression of HACD2, PKM2, and c‐Myc after HACD2 knockdown in vivo. G) Representative IHC staining of HACD2, PKM2, and c‐Myc in xenograft tissues after HACD2 knockdown in vivo. Scale bars, 100 µm. H) Representative immunofluorescence staining of HACD2 and PKM2 in tissues from PC patients. Scale bars, 50 µm. I) The ECAR was measured in cells transfected with shHACD2, shNC, shHACD2 + HACD2^WT^, or shHACD2 + HACD2^MUT^ via an XF Extracellular Flux Analyzer, *n* = 4. J) Statistical analysis of the effects of HACD2 replenishment on glycolytic activity, *n* = 4. Data were presented as means ± SD. ***p < 0.001 according to one‐way ANOVA.

To explore the mechanism by which HACD2 regulates PC cells proliferation, we performed gene set enrichment analysis (GSEA) of the DEGs, and the results revealed that the glycolytic pathway was activated after HACD2 overexpression (**Figure** **3**A). Compared with the control cells, the HACD2‐silenced cells presented lower extracellular acidification rate (ECAR) values (Figure 3B). Consequently, HACD2 knockdown inhibited glycolysis while reducing the glycolytic capacity and the glycolytic reserve in AsPC‐1 and PANC‐1 cells (Figure 3B,C). Moreover, the glucose uptake capacity and extracellular lactate levels of pancreatic cancer cells decreased after HACD2 knockdown, whereas HACD2 overexpression had the opposite effect (Figure S4A,B, Supporting Information). Gene Ontology (GO) enrichment analysis of the HACD2‐interacting proteins also revealed enrichment in the carbon metabolic pathway (Figure S4C, Supporting Information). In addition, lactate levels in the tumor tissues of the HACD2‐knockdown tumor‐bearing mice were decreased (Figure 3D), suggesting that HACD2 is indeed involved in the glycolytic process in PC.

To further elucidate the mechanism by which HACD2 regulates glycolysis, we performed GEPIA correlation analysis, which revealed that the HACD2 mRNA expression levels were consistent with those of key enzymes (GLUT1(Solute carrier family 2, facilitated glucose transporter member 1), PFKFB3 (6‐phosphofructo‐2‐kinase/fructose‐2,6‐bisphosphatase 3), PKM2) in the glycolytic pathway (Figure S4D, Supporting Information). Moreover, the expression levels of key enzymes in the glycolytic pathway were determined via RT‒qPCR and western blotting. Interestingly, the mRNA levels of PKM2 were not substantially affected by HACD2 knockdown or overexpression, whereas its protein expression mirrored that of HACD2, and PFKFB3 was not significantly affected (Figure 3E; and Figure S4E, Supporting Information). Similar results were obtained via western blotting and immunohistochemical analysis of tumor tissues from PANC‐1 tumor‐bearing mice, which revealed that HACD2 knockdown decreased PKM2 expression (Figure 3F,G). Immunofluorescence staining of tumor tissue from clinical patients also confirmed that the expression levels of HACD2 and PKM2 tended to be consistent (Figure 3H). Moreover, both wild‐type HACD2 (HACD2^WT^) and enzymatically mutated HACD2 (HACD2^MUT^) reversed the decrease in glycolysis in PC cells caused by HACD2 knockdown (Figure 3I,J). Overall, HACD2 promoted glycolysis in PC cells, possibly by increasing the expression of PKM2.

### 2.4 HACD2 Knockdown Increases PKM2 Ubiquitination in a Nonenzymatic Manner

**Figure 4:**
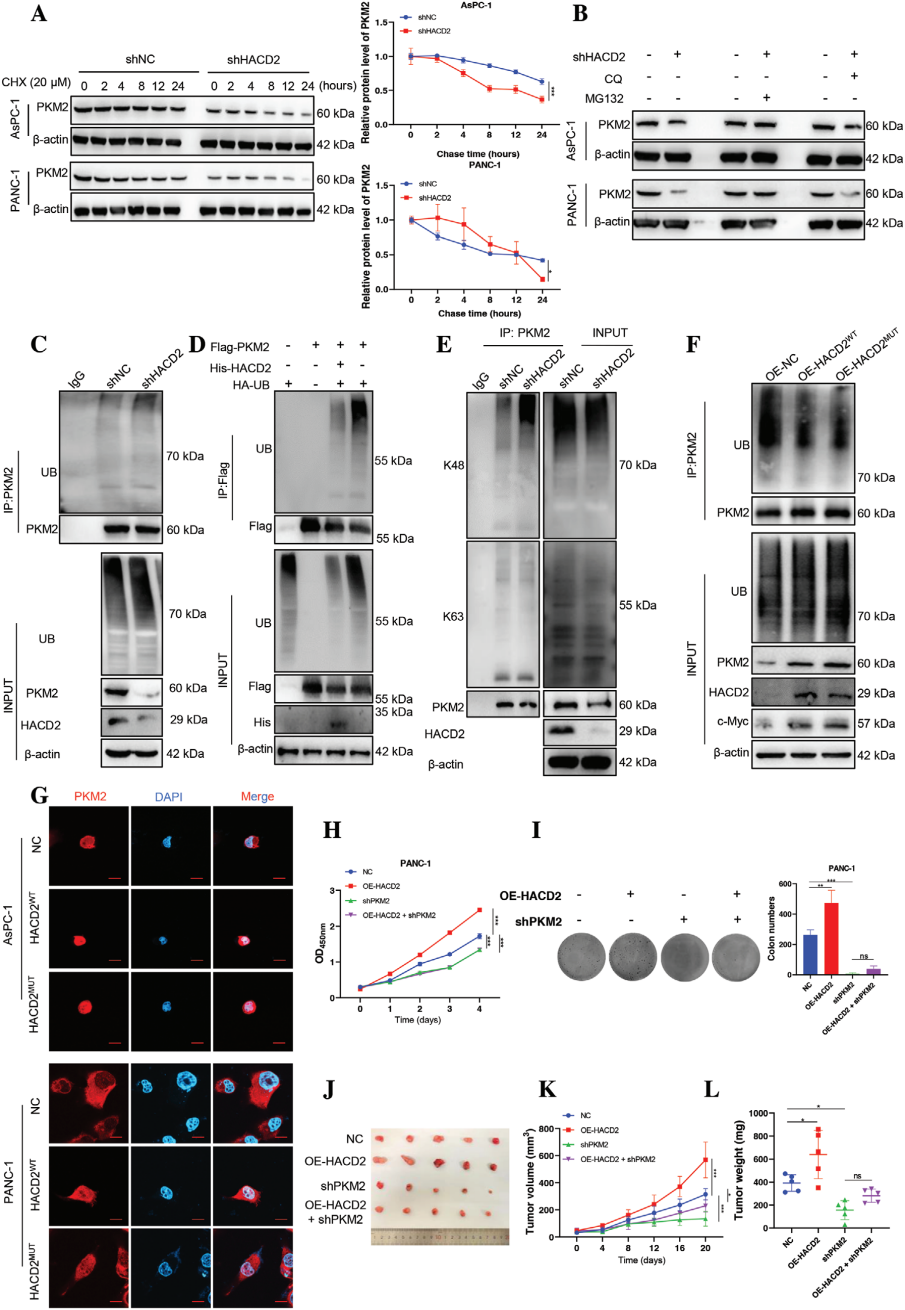
HACD2 knockdown increases PKM2 ubiquitination in a nonenzymatic manner. A) The degradation of the PKM2 protein in HACD2‐knockdown PC cells was measured by cycloheximide chase assays. Data were presented as means ± SD. *p < 0.05, ***p < 0.001 according to two‐way ANOVA, *n* = 3. B) HACD2‐deficient PC cells were treated with MG132 or CQ, and PKM2 protein levels were assessed by western blotting. C) Ubiquitination of PKM2 in PC cells treated for 8 h with 10 µm MG132 after HACD2 knockdown, as detected by immunoprecipitation. D) The indicated HEK‐293T cells were transfected with plasmids expressing HA‐UB, Flag‐PKM2, or His‐HACD2, and the cell lysates were immunoprecipitated with an anti‐Flag antibody, followed by western blotting. E) PANC‐1 cell lysates were immunoprecipitated with an anti‐PKM2 antibody to detect the linkage‐specific ubiquitin ligases for PKM2 after HACD2 knockdown. F) PKM2 monomers and dimers in AsPC‐1 and PANC‐1 cells transduced with HACD2^WT^, HACD2^MUT^, or a vector control were detected by western blotting. G) The nuclear translocation of PKM2 in control, HACD2^WT^ and HACD2^MUT^ AsPC‐1 and PANC‐1 cells was assessed by immunofluorescence. Scale bar, 20 µm. H,I) CCK‐8 (*n* = 4) and colony formation (*n* = 3) assays showing the effects of PKM2 knockdown on cell proliferation after HACD2 overexpression. Data were presented as means ± SD. ns., not significant; **p < 0.01, ***p < 0.001 according to two‐way ANOVA and one‐way ANOVA. PANC‐1‐related stable cells (control, HACD2, shPKM2, and HACD2 + shPKM2) were injected into the right flanks of null mice. The tumor volumes were measured every 4 days. J–L) Tumor images, growth curves and weights were obtained after dissection on day 20, *n* = 5. Data were presented as means ± SD. *p < 0.05, **p < 0.01, ***p < 0.001 according to two‐way ANOVA and one‐way ANOVA.

Previous results have shown that HACD2 may play an important role in the protein stability of PKM2 (Figure 3E; and Figure S4E, Supporting Information). Cycloheximide (CHX) chase assays revealed that HACD2 knockdown significantly enhanced the PKM2 degradation and impaired its protein stability (**Figure** **4**A). To further investigate the molecular mechanism by which HACD2 regulates PKM2, we treated HACD2‐knockdown PC cells with a ubiquitin‐proteasome inhibitor (MG132) or an autophagy‐lysosomal inhibitor (CQ), and the protein expression level of PKM2 was not rescued after HACD2 knockdown in the presence of CQ; however, this inhibitory effect on protein expression was abolished in the presence of MG132 (Figure 4B), indicating that the effect of HACD2 on PKM2 stability is mediated through the ubiquitin‐proteasome pathway. Consistent with this result, HACD2 knockdown increased the level of ubiquitinated PKM2, whereas HACD2 overexpression decreased the level of ubiquitinated PKM2 (Figure 4C,D). Full‐length ubiquitin contains seven lysine sites (K6, K11, K27, K29, K33, K48, and K63), of which K48 and K63 are the two most common ubiquitin‐linked chains.^[^ ^24^ ^]^ To identify the ligase by which HACD2 regulates PKM2 ubiquitination, we further measured the PKM2 K48 and K63 levels via immunoprecipitation. The PKM2 K48 level was increased, and the K63 level was barely changed in the HACD2‐knockdown cells (Figure 4E). These results indicated that HACD2 knockdown deregulates PKM2 protein levels by increasing its ubiquitination.

Protein dimerization and oligomerization have been shown to be key steps in the activation of PKM2.^[^ ^25^ ^]^ The expression of the PKM2 dimer after HACD2 knockdown was reduced (Figure S5A, Supporting Information). Western blotting and immunofluorescence demonstrated that the deletion of HACD2 blocked the nuclear translocation of PKM2 and promoted the expression of the myelocytomatosis viral oncogene homolog (c‐Myc), whereas the overexpression of HACD2 had the opposite effect (Figure S5B–D, Supporting Information). Additionally, treatment with MG132 reversed the decrease in PKM2 dimerization and its movement to the nucleus following HACD2 knockdown (Figure S5A–C, Supporting Information). These findings indicated that the HACD2‐mediated nuclear translocation and dimerization of PKM2 rely on the ubiquitin‐proteasome pathway.

We demonstrated that the proliferative effect of HACD2 on PC is not dependent on dehydratase function, as shown in Figure 2. To exclude the effect of HACD2 enzymatic activity on the stability of the PKM2 protein, we incubated PC cells with VLCFAs after HACD2 knockdown. As expected, VLCFAs did not block the effects of HACD2 knockdown on the PKM2 mRNA expression level and ubiquitination (Figure S6A,B, Supporting Information). Consistent with this finding, CHX chase assays revealed that both HACD2^WT^ and HACD2^MUT^ could increase PKM2 protein stability (Figure S6C, Supporting Information), and HACD2^MUT^ decreased ubiquitination of PKM2 as same as HACD2^WT^ (Figure 4F). In other words, the ability of HACD2 to regulate the stability of PKM2 is independent of its dehydratase properties. Moreover, the replenishment of VLCFAs failed to increase PKM2 dimer after HACD2 knockdown (Figure S6D, Supporting Information). The immunofluorescence and western blot results confirmed that compared with HACD2^WT^, HACD2^MUT^ did not affect the dimer formation or nuclear translocation of PKM2, and the protein expression levels of c‐Myc and GLUT1 regulated by HACD2^MUT^ were consistent with those of HACD2^WT^ (Figure 4G; and Figure S6E, Supporting Information). These findings indicated that HACD2 regulates PKM2 dimerization and nuclear translocation independent of its enzymatic activity.

To further investigate the role of HACD2 in regulating the growth of PC through PKM2, we generated HACD2‐overexpression and PKM2‐knockdown cell lines to validate the regulation of tumors by the HACD2/PKM2 axis (Figure S6F, Supporting Information). As expected, CCK‐8 and plate cloning assays revealed that the proliferation of PC cells in which HACD2 was overexpressed was abolished by PKM2 knockdown (Figure 4H,I). Similar results were obtained for subcutaneous xenograft tumors (Figure 4J–L), suggesting that PKM2 knockdown blocked the excessive proliferation of PC cells caused by HACD2 overexpression. Overall, these results suggested that HACD2 regulates the ubiquitination and dimerization of PKM2 for nuclear translocation in an enzyme‐independent manner to promote PC progression.

### 2.5 HACD2 Directly Binds to PRKN and Enhances the Dissociation of PKM2 from PRKN

**Figure 5:**
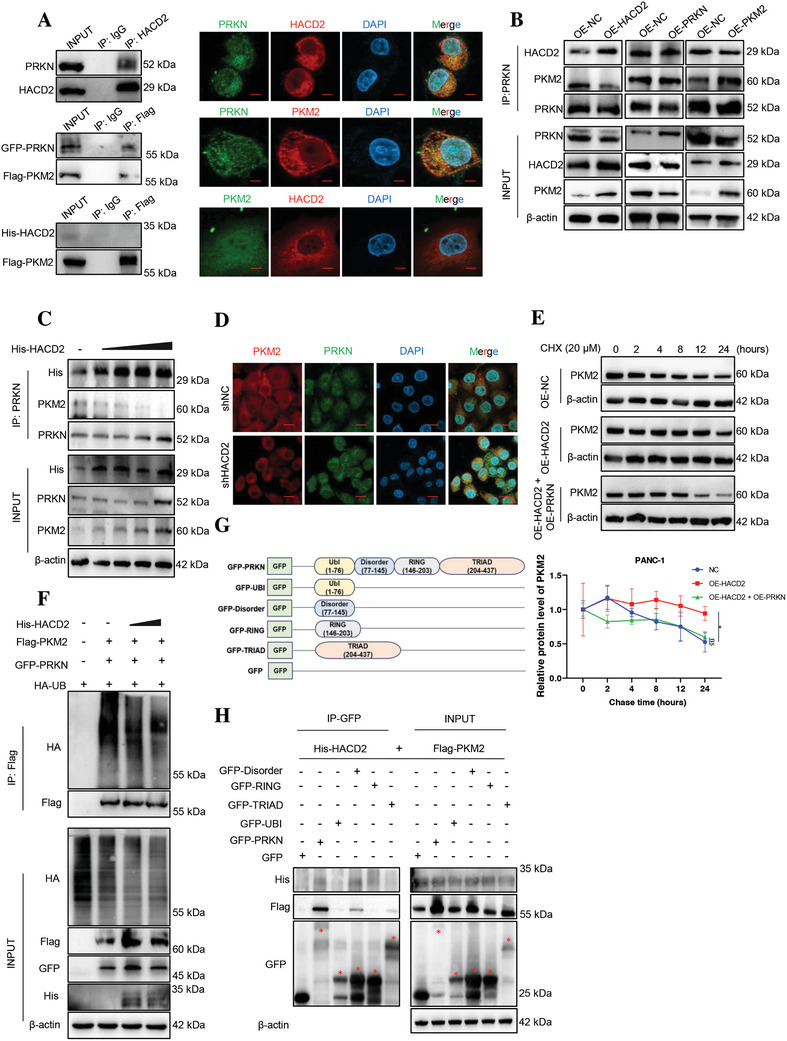
HACD2 directly binds to PRKN and enhances the dissociation of PKM2 from PRKN. A) The interaction between HACD2 and PRKN was examined by immunoprecipitation and immunofluorescence assays in PANC‐1 cells (top). The interaction between PRKN and PKM2 was examined via an immunoprecipitation assay in HEK‐293T cells and an immunofluorescence assay in PANC‐1 cells (middle). The interaction between HACD2 and PKM2 was examined by an immunoprecipitation assay in HEK‐293T cells and an immunofluorescence assay in PANC‐1 cells (bottom). Scale bar, 10 µm. B) PRKN was immunoprecipitated from PANC‐1 cells transduced with HACD2, PKM2, PRKN, or a vector control and analyzed by western blotting to assess the binding of endogenous PKM2 and HACD2 to PRKN. C) His‐HACD2 was immunoprecipitated from PANC‐1 cells in a dose‐dependent manner via an anti‐PRKN antibody and analyzed by western blotting. D) Immunofluorescence was used to assess the colocalization of PRKN and PKM2 after HACD2 overexpression in PANC‐1 cells. Scale bar, 20 µm. E) Degradation of the PKM2 protein in PANC‐1 cells overexpressing HACD2 or PRKN was measured by cycloheximide chase assessment, *n* = 3. Data were presented as means ± SD. ns., not significant; *p < 0.05 according to two‐way ANOVA. F) Immunoprecipitation was used to assess the effect of PRKN on PKM2 ubiquitination. G) Schematic diagram of the GFP‐PRKN structural domain deletion construct. H) The interaction between PRKN truncated and HACD2 or PKM2 was detected by immunoprecipitation in HEK‐293T cells.

To investigate the mechanism by which HACD2 regulates the stability of PKM2, we identified proteins that interact with HACD2 via immunoprecipitation‐mass spectrometry (IP‐MS). PRKN not only interacts with HACD2, but also interacts with PKM2 (Figure S7A, Supporting Information). Immunofluorescence and coimmunoprecipitation again demonstrated that PRKN could bind to both PKM2 and HACD2, and there was no direct binding between HACD2 and PKM2 (**Figure** **5**A). These findings suggested that PRKN may act as a link between HACD2 and PKM2 in PC cells.

Immunoprecipitation revealed that high levels of HACD2 blocked the interaction between PKM2 and PRKN; high levels of PKM2 further promoted the binding between PKM2 and PRKN; and high levels of PRKN did not affect the binding of PKM2 and HACD2 to PRKN (Figure 5B; and Figure S7B, Supporting Information), suggesting that HACD2 may disrupt the interaction between PRKN and PKM2. Therefore, we overexpressed PRKN in addition to HACD2 and found that the balance of PRKN binding between PKM2 and HACD2 was disrupted (Figure S7C, Supporting Information). This interference effect was observed in a HACD2 dose‐dependent manner (Figure 5C; and Figure S7D, Supporting Information), a finding that was similar to the results of the immunofluorescence experiments showing that the presence of HACD2 may interfere with the interaction between PRKN and PKM2, thereby stabilizing PKM2 expression in PC cells and indicating that HACD2 directly binds to PRKN and enhances the dissociation of PKM2 from PRKN (Figure 5D). CHX chase assays further confirmed that HACD2 overexpression weakened the PKM2 degradation caused by PRKN overexpression (Figure 5E). Moreover, immunoprecipitation revealed that the presence of PRKN reversed the reduced ubiquitination of PKM2 caused by HACD2 overexpression (Figure 5F), and the binding of PRKN to HACD2 also promoted the ubiquitination of HACD2 (Figure S7E, Supporting Information). On the basis of the domain characteristics of PRKN, we constructed four truncations of PRKN (UBI, Disorder, RING, and TRIAD). The immunoprecipitation results revealed that the “Disordered” region of PRKN was the major region where PRKN induced competitive binding between PKM2 and HACD2 (Figure 5G,H). These results revealed that HACD2 can decrease PKM2 ubiquitination by directly binding to PRKN and dissociating PKM2 from the “Disordered” regions of PRKN.

### 2.6 PRKN Overexpression Blocks HACD2‐Induced PC Growth

**Figure 6:**
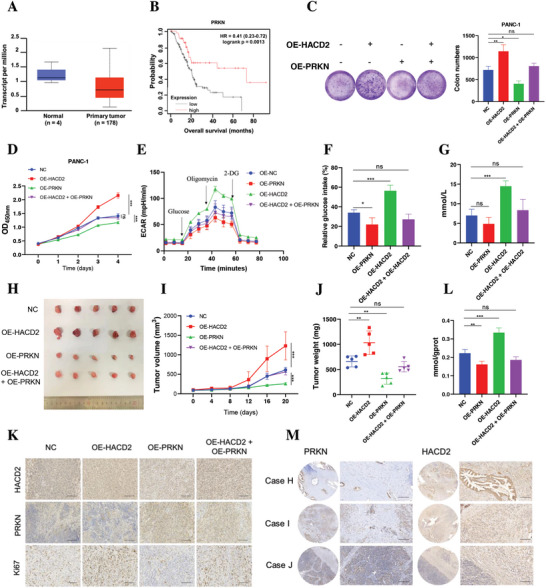
PRKN overexpression blocks HACD2‐induced PC growth. A) The differential expression of PRKN transcripts in pancreatic cancer and normal tissues was analyzed via the UALCAN database (https://ualcan.path.uab.edu/). B) Using an online tool, Kaplan–Meier analysis revealed that PRKN expression levels are correlated with poor overall survival and progression‐free survival in pancreatic cancer patients via a Kaplan‒Meier plotter database (https://kmplot.com/analysis/). C,D) Colony formation assays (*n* = 3) and CCK‐8 (*n* = 4) assays were used to assess cell viability and proliferation after HACD2 and PRKN overexpression in PANC‐1 cells. Data were presented as means ± SD. ns., not significant; *p < 0.05, **p < 0.01 according to one‐way ANOVA and two‐way ANOVA. PANC‐1‐related stable cells (control, HACD2, PRKN, and HACD2+PRKN) were injected into the right flanks of null mice. E) ECAR was measured in cells transfected with shHACD2 or shNC via an XF Extracellular Flux Analyzer. *n* = 4. F,G) The relative glucose intake and lactate release by PC cells with HACD2 knockdown or overexpression were monitored. *n* = 4. Data were presented as means ± SD. ns., not significant; *p < 0.05, **p < 0.01, ***p < 0.001 according to one‐way ANOVA. H–J) Tumor volume was measured every 4 days. *n* = 5. Tumor images, growth curves, and weights were obtained after dissection on day 20. Data were presented as means ± SD. **p < 0.01, ***p < 0.001 according to two‐way ANOVA and one‐way ANOVA. K) Representative IHC staining of HACD2, PRKN, and Ki67 in xenograft tissues after HACD2 or PRKN overexpression in vivo. Scale bars, 100 µm. L) Relative lactate release in tumor tissue from PANC‐1 tumor‐bearing mice (OE‐NC, OE‐HACD2, OE‐PRKN, and OE‐HACD2 + OE‐PRKN). *n* = 4. Data were presented as means ± SD. ns., not significant; *p < 0.05, according to one‐way ANOVA. M) Representative IHC staining of PKM2, HACD2, and PRKN in pancreatic cancer tissues from patients. Scale bars, 100 µm.

As a tumor suppressor gene, PRKN is expressed at low levels in PC and is associated with poor prognosis (**Figure** **6**A,B). Moreover, immunohistochemistry results indicated that the expression of PRKN in adjacent tissues was substantially greater than that in PC tissues (Figure S8A, Supporting information). Additionally, western blot analysis revealed that PRKN levels in tumor cells were lower than those in normal pancreatic epithelial cells (Figure S8B, Supporting information). We next investigated whether PRKN could block HACD2‐mediated tumor growth. Stable PC cell lines overexpressing PRKN and HACD2 were constructed (Figure S8C, Supporting Information). In vitro, PRKN overexpression inhibited the proliferation of PC cells, and blocked the proliferation of PC cells caused by HACD2 overexpression (Figure 6C,D). Moreover, PRKN overexpression reversed the increase in glycolysis induced by HACD2 overexpression in PC cells (Figure 6E–G). Consistent with the in vitro results, PRKN overexpression similarly blocked HACD2‐induced proliferation of subcutaneously transplanted tumors (Figure 6H–K), and the increase in lactate levels caused by HACD2 overexpression in tumor tissues was also alleviated by PRKN overexpression (Figure 6L). These results suggested a negative regulatory relationship between HACD2 and PRKN in PC. Immunohistochemical staining of tumor tissues from clinical patients with PC verified the negative correlation between HACD2 and PRKN expression (Figure 6M). Overall, PRKN overexpression repressed HACD2‐induced PC growth.

### 2.7 Orlistat Inhibits PC Growth by Targeting HACD2

**Figure 7:**
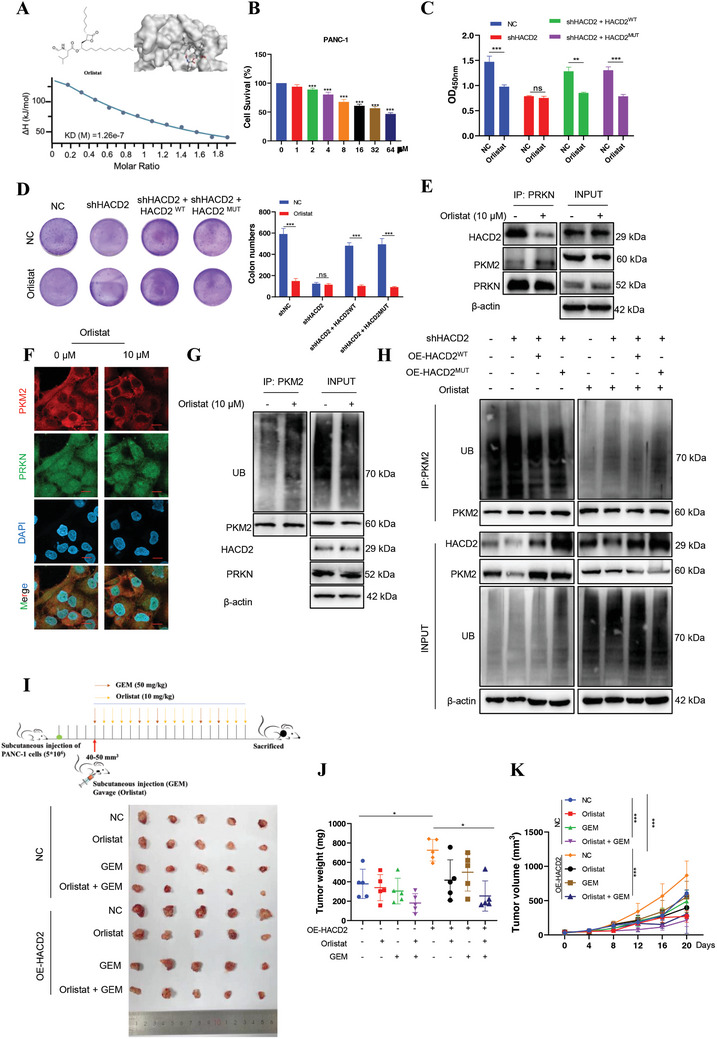
Orlistat inhibits PC growth by targeting HACD2. A) Isothermal titration calorimetry analysis of orlistat affinity (bottom) and the docking pocket for HACD2 (top). B) Sensitivity of PANC‐1 cells to orlistat. *n* = 4. Data were presented as means ± SD. ***p < 0.001 according to one‐way ANOVA. C,D) The sensitivity of orlistat to PANC‐1 cells with HACD2 knockdown or complemented active site mutation to orlistat was detected by CCK‐8 (*n* = 4) and plate cloning (*n* = 3). Data were presented as means ± SD. ns., not significant; **p < 0.01, ***p < 0.001 according to unpaired two‐tailed Student's *t*‐test. E) Immunoprecipitation was used to assess the interaction of HACD2, PKM2, and PRKN in PC cells after treatment with 10 µm orlistat. F) Immunofluorescence was used to assess the interaction between PKM2 and PRKN after treatment with 10 µm orlistat in PANC‐1 cells. Scale bar, 20 µm. G) The ubiquitination of PKM2 was measured by immunoprecipitation after 10 µm orlistat treatment in PANC‐1 cells. H) The ubiquitination of PKM2 in the HACD2 enzyme activity locus mutant in PANC‐1 cells was measured via immunoprecipitation after orlistat treatment. I–K) PANC‐1‐related stable cells (control, OE‐HACD2) were injected into the right flanks of null mice. The tumor volumes were measured every 4 days. *n* = 5. Tumor images, tumor weight and growth curves were obtained after the intraperitoneal injection of 50 mg kg^−1^ gemcitabine twice a week and the intragastric administration of 10 mg kg^−1^ of orlistat five times a week. Data were presented as means ± SD. *p < 0.05, ***p < 0.001 according to two‐way ANOVA.

HACD2 plays an important role in regulating the proliferation of PC cells. However, there is no known inhibitor that targets HACD2. Therefore, we speculated that a specific antiobesity drug could effectively inhibit the proliferation of PC cells. Isothermal titration calorimetry (ITC) verified the interaction between HACD2 and orlistat (*K*_D_ = 0.13 µm) (**Figure** **7**A). Orlistat does not act on the central nervous system; it mainly inhibits lipase in the human gastrointestinal tract and reduces fat intake to achieve weight control. CCK‐8 assays confirmed that orlistat had a good inhibitory effect on PC cells (Figure 7B). In addition, HACD2 knockdown successfully blocked the inhibitory effect of orlistat on the proliferation of PC cells, and the inhibitory effect on PC cells was rescued after the replacement of HACD2^WT^ or HACD2^MUT^ (Figure 7C,D). These results demonstrated that the antitumor effect of orlistat did not depend on HACD2 enzymatic activity. Moreover, the binding of PRKN to PKM2 increased and that to HACD2 decreased in PC cells after treatment with 10 µm orlistat for 48 h, as shown by immunoprecipitation and immunofluorescence analyses (Figure 7E,F). The ubiquitination of PKM2 increased after orlistat treatment (Figure 7G), and the HACD2 and PRKN protein expression levels were not affected by orlistat (Figure S9A, Supporting Information). These findings indicated that orlistat can bind to HACD2 and release PRKN, thereby binding to PKM2 and promoting its degradation. Moreover, the immunoprecipitation results indicated that the effect of orlistat on PKM2 ubiquitination was independent of the dehydratase activity of HACD2 (Figure 7H). In the HACD2‐overexpressing tumor‐bearing mice, the rate of orlistat inhibition reached 31.3% (Figure 7I–K). The above results indicated that orlistat inhibits the expression of PKM2 and blocks the proliferation of PC cells by binding to HACD2 and releasing PRKN to bind to PKM2.

To explore the binding sites between HACD2 and orlistat, we predicted the interaction sites between HACD2 and orlistat via the Molecular operating environment (MOE) and found that the 165 and 242 sites had binding potential (Figure S9B, Supporting Information). The triglyceride (TG) content and Oil Red O staining results revealed that single or double mutation at positions 165 and 242 did not affect the enzymatic activity of HACD2 (Figure S9C,D, Supporting Information). However, the inhibitory effect of orlistat on PC cells proliferation was blocked by the double mutation (Figure S9E,F, Supporting Information). The results of immunoprecipitation also revealed that the double‐site mutation of HACD2 could inhibit the ubiquitination effect of orlisitat on PKM2, thereby blocking its degradation (Figure S9G, Supporting Information). The above preliminary results demonstrated that the orlistat can bind to the 165 and 242 loci of HACD2 and play an antitumor role.

## 3 Discussion

HACD2 has been increasingly reported in the literature as a tumor marker,^[^ ^16^, ^26^, ^27^ ^]^ and HACD2 deficiency can cause angiogenic arrest and further lead to embryonic lethality.^[^ ^17^ ^]^ However, its biological function and mechanism of action in tumors are still unclear. In our study, HACD2 was highly expressed in PC, especially in the advanced‐stage PC, and was correlated with patient prognosis (Figure 1A–D; and Table S1, Supporting Information), and HACD2 knockdown effectively inhibited the progression of PC (Figure 1E–K). Taken together, our results clearly demonstrated that HACD2 plays a non‐negligible role as an oncogene in PC progression. This study also provides a new direction for the development of clinical PC drugs. However, the reason HACD2 is upregulated in PC needs to be further explored.

PC is a highly malignant and fatal disease caused by a complex tumor microenvironment and abnormal metabolic reprogramming.^[^ ^28^ ^]^ Glucose metabolism and lipid metabolism are closely related during tumor development. Previous studies have shown that HACD2, an obesity‐related gene, plays an important role in the regulation of fatty acid synthesis.^[^ ^12^ ^]^ Surprisingly, in our study, the replenishment of long‐chain fatty acids did not rescue the inhibition of PC cells proliferation caused by HACD2 deletion (Figure 2), suggesting that the regulatory effect of HACD2 on the proliferation of PC cells is dependent on its nonenzymatic function. In the classic signaling pathway, HACD2 mainly regulates the dehydration step during the elongation of VLCFAs, promoting the dehydration of 3‐hydroxyacyl‐CoA intermediates to trans‐2,3‐enoyl‐CoA.^[^ ^10^, ^29^ ^]^ However, in this study, we elucidated a novel role for HACD2 in the regulation of glucose metabolism in PC to promote the proliferation of PC cells. In fact, many enzymes in metabolic pathways can not only exert their own enzymatic activity but also impart nonenzymatic activity to exert their function. For example, cytoplasmic PKM2 catalyzes glycolysis, and nuclear PKM2 activates TMEM33 to promote lipid synthesis.^[^ ^30^ ^]^ PCK1, the key enzyme in the gluconeogenesis pathway, can also promote lipid synthesis in tumors by activating INSIG1/2.^[^ ^20^ ^]^ In other words, glucose and lipid metabolism in tumors are often mutually affected, and enzymes in specific pathways may play important roles in other metabolic pathways. Our reasonable inference was that the functional regulation of metabolic enzymes during disease progression comes not only from the regulation of their classic activities in certain pathways but also from the regulation of their nonclassic or nonmetabolic activities. The results of this study provided new ideas and insights for subsequent research on metabolic pathways.

PKM2 is a rate‐limiting enzyme that regulates the last step of the glycolytic pathway.^[^ ^31^ ^]^ In vitro, PKM2 exists mainly in a tetrameric form that has pyruvate kinase activity and in a dimeric form that localizes to the nucleus to have protein kinase activity.^[^ ^32^ ^]^ Under the stimulation of epidermal growth factor, PKM2 undergoes *cis*‐*trans* isomerization to expose a nuclear localization signal and then binds to importin‐5 for nuclear transport.^[^ ^28^, ^33^, ^34^, ^35^ ^]^ In addition, the hydroxylation or binding of PKM2 to bifunctional peptidase and arginyl‐hydroxylase can increase its nuclear translocation.^[^ ^36^ ^]^ According to previous studies, PKM2 is degraded mainly by binding to heat shock proteins in a lysosome‐dependent manner via molecular chaperone‐mediated autophagy. In our study, the presence of HACD2 resulted in an increase in the level of the PKM2 dimer and its nuclear transport (Figure 4F,G; and Figure S5, Supporting Information). However, the downregulation of PKM2 induced by HACD2 knockdown could not be reversed by supplementation with an autophagy inhibitor, and the degradation of PKM2 was blocked by the addition of a proteasome inhibitor (Figure 4B), indicating that HACD2‐mediated PKM2 degradation was dependent on the ubiquitin‐protein proteasome. Similarly, a previous report showed that PKM2 is degraded under the regulation of DDX39 by the ubiquitin‐proteasome system.^[^ ^37^ ^]^ Thus, our study identified a special degradation mode of PKM2; the mode of protein degradation in vivo is not unique and is often affected by multiple regulatory factors. In addition, the mRNA and protein levels of GLUT1, a key enzyme in the glycolytic pathway, were significantly altered in our study, but we did not investigate this further because c‐Myc has been reported to act as a transcriptional regulator of GLUT1 to promote its mRNA expression.^[^ ^38^ ^]^ Despite the notable antitumor effects of PKM2, it is difficult to directly target PKM2 in tumors. Therefore, it is necessary to explore its upstream and downstream targets.

Furthermore, current studies have shown that HACD2 promotes PC progression by binding to PRKN and dissociating PKM2 from PRKN (Figure 5), decreasing PKM2 ubiquitination and leading to tumorigenesis and that this phenomenon is inhibited by PRKN overexpression (Figure 6). In other words, competitive binding of PRKN between HACD2 and PKM2 occurred. As a recessive gene involved in the pathogenesis of Parkinson's disease, PRKN can affect mitochondrial function, calcium homeostasis, synaptic function, and lysosomal/proteasome degradation.^[^ ^39^, ^40^, ^41^ ^]^ In addition, PRKN plays an important role in tumorigenesis, and abnormal expression of PRKN can lead to uncontrolled growth and proliferation, inhibit cell death and induce angiogenesis. In recent years, many neuropathy‐related disease targets have been reported to be related to the development of tumors,^[^ ^42^ ^]^ suggesting that tumor suppression can be achieved by regulating the nervous system.

Orlistat is a weight‐loss drug that prevents the hydrolysis of triglycerides by inhibiting gastrointestinal fatty acid synthesis. Previous studies have shown that orlistat can effectively inhibit the proliferation, invasion, and migration of colon cancer cells.^[^ ^43^, ^44^ ^]^ In our study, orlistat interfered with the interaction between HACD2 and PRKN by binding to HACD2 (*K*_D_ = 0.13 µm), which further inhibited the expression of PKM2 and c‐Myc and reduced the proliferation of PC cells, especially in combination with gemcitabine (GEM) (Figure 7). Furthermore, the target specificity of small‐molecule drugs is not particularly robust, indicating that more research is necessary moving forward. Given the complex changes in the tumor microenvironment of PC, further verification of the efficacy of drug combinations, such as experiments utilizing orthotopic transplantation of tumors and patient‐derived tumor xenografts, is necessary. Therefore, as an obesity‐related gene, HACD2 could be a potential target for inhibiting PC progression clinically.

## 4 Conclusions

**Figure 8:**
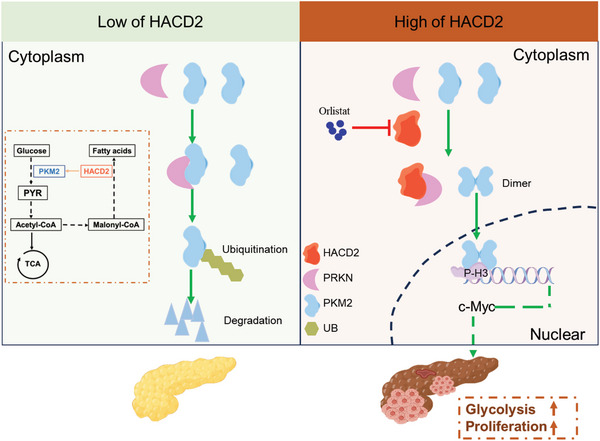
Schematic illustration of the proposed model in which the HACD2/PRKN/PKM2 axis regulates PC tumor growth. HACD2 promotes the progression of pancreatic cancer by dissociating PKM2 from PRKN, resulting in a decrease in PKM2 ubiquitination and an increase in PKM2 dimerization, followed by the promotion of c‐Myc expression and tumor proliferation. In addition, orlistat disrupted the binding of HACD2 and PRKN, increasing PKM2 ubiquitination.

Overall, we found that high HACD2 expression promoted the proliferation of PC cells in a nonenzymatic manner. Mechanistic studies revealed that the protumorigenic effect of HACD2 occurred through the inhibition of the proteasome‐dependent ubiquitination of PKM2 and the activation of PKM2 dimerization, leading to c‐Myc expression (**Figure** **8**). In addition, orlistat potentially binds to HACD2, disrupting the interaction between HACD2 and PRKN and further increasing the ubiquity of PKM2. These results strongly suggested that HACD2 plays a role in the progression of PC and can be used as a therapeutic target for this disease.

## 5 Experimental Section

### Regents

Doxycycline (Dox), VLCFAs, AH‐7614, MG132, CQ, and orlistat were purchased from MedChemExpress (Shanghai, China). Glucose detection kit, LD Assay Kit, TG Assay Kit were purchased from Jiancheng Bioengineering Institute (Nanjing, Jiangsu, China); DAPI, Oil‐Red‐O, 4% paraformaldehyde (PFA), Nonidet P 40 (NP‐40), and Phenylmethanesulfonyl fluoride (PMSF) were purchased from Beyotime Institute of Biotechnology (Nanjing, Jiangsu, China).

### Cell Culture

The pancreatic cell lines PANC‐1 and AsPC‐1 and the human embryonic cell line HEK‐293T were obtained from the Cell Bank of the Chinese Academy of Science. The cells were cultured in Dulbecco's modified eagle medium (DMEM, Gibco, Grand Island, NY) supplemented with 10% foetal bovine serum (FBS, Vazyme, Nanjing, Jiangsu, China) and 1% penicillin/streptomycin (Beyotime Institute of Biotechnology, Nanjing, Jiangsu, China) at 37 °C with 5% CO_2_ in a humidified incubator.

### Human Sample Collection

All relevant ethical regulations were followed in this study. Human tissue samples were obtained from the Department of General Surgery, Rui Jin Hospital, Research Institute of Pancreatic Disease, School of Medicine, Shanghai JiaoTong University. Furthermore, 83 pairs of PC tissue chips were obtained from the Department of General Surgery at Rui Jin Hospital, Research Institute of Pancreatic Disease, School of Medicine, Shanghai JiaoTong University, to examine the relationships between HACD2 and factors such as patient age, gender, stage, and survival. All patients signed an informed consent form approved by the Ethics Committee of the Department of General Surgery, Ruijin Hospital.

### Animal Experiments

The animal experiments were approved by the Institutional Animal Care and Use Committee of China Pharmaceutical University. Four‐week‐old female BALB/c nude mice were purchased from Yangzhou University, Jiangsu, China. All the mice were housed at 22 °C with a 12 h light/dark cycle and unlimited access to water and food.

For the subcutaneous tumor model, the indicated PANC‐1 cells (5 × 10^6^ cells in 200 µL of PBS per mouse) were injected into the left underarm of the mice, and the volume of the axillary tumors in the mice was measured every 4 days. PANC‐1 tumor‐bearing mice were treated intraperitoneally with GEM every two times a week or intragastrically with orlistat 5 days a week. The mice were sacrificed after 20 days, and the tumors were removed, weighed, and subjected to IHC. For orthotopic tumor transplantation, PANC‐1 cells (1 × 10^7^ cells in 100 µL of PBS per mouse) were inoculated into the mouse pancreas. Eight weeks later, the mice were sacrificed, and the pancreata was removed, weighed, and subjected to IHC.

### Immunoprecipitation

The treated cells were washed with precooled PBS, and then NP‐40 lysis buffer containing 1% PMSF was added for lysis. The cells were then scraped off the plate with a spatula and incubated on ice for 30 min (vortexing every 10 min). After centrifugation at 12 000 rpm for 15 min at 4 °C, the supernatant was collected, and antibodies were added, followed by incubation at 4 °C overnight. The treated magnetic beads were added, and the samples were incubated at 4 °C for 4 h. Finally, the beads were washed and analyzed by western blotting.

### Immunofluorescence

The cells on the climbs were washed three times with PBS and then fixed with 4% paraformaldehyde (PFA) for 30 min, followed by 30 min of membrane disruption after three washes with PBS and the addition of 0.2% Triton X‐100. After washing with PBS for three times. This was followed by three more washes with PBS, 5% BSA was added for blocking for 30 min, and finally, primary antibodies were added and incubated overnight at 4 °C. The primary antibody was removed, and the fluorescein secondary antibody was added and incubated at room temperature for 3 h. After three washes with PBS, DAPI was added, and the samples were stained for 30 min at room temperature. Finally, the samples were observed under a laser confocal microscope (LSM800).^[^ ^45^ ^]^

### Western Blotting

The treated cells were lysed with RIPA buffer, and the lysed cells were collected and centrifuged at 12 000 g for 15 min at 4 °C. The supernatant was subsequently collected, and the protein concentration was determined via a BCA kit (Vazyme, Nanjing, Jiangsu, China). After electrophoresis through polyacrylamide gels and transfer of proteins to PVDF membranes (Millipore Corp, Billerica, MA), the cells were blocked with 5% milk for 2 h at room temperature. The PVDF membranes were then incubated with primary antibodies against HACD2 (Cusabio, Wuhan, Hubei, China, 1:2000), PKM2 (ZEN‐BIOSCIENCE, Chengdu, Sichuan, China, 1:2000), PRKN (HuaBio, Hangzhou, Zhejiang, 1:1000), c‐Myc (ZEN‐BIOSCIENCE, Chengdu, Sichuan, 1:2000), LDHA (ZEN‐BIOSCIENCE, Chengdu, Sichuan, China, 1:1000), GLUT1 (ZEN‐BIOSCIENCE, Chengdu, Sichuan, China, 1:1000), PFKFB3 (ZEN‐BIOSCIENCE, Chengdu, Sichuan, China, 1:1000), and β‐actin (ABclone Technology, Beijing, China, 1:10 000) diluted in 1% BSA overnight at 4 °C. Finally, the PVDF membranes were incubated with the secondary antibody (HuaBio, Hangzhou, Zhejiang, 1:10 000) for 2 h at room temperature. The final results were visualized by chemiluminescence.

### RT‒qPCR

Total RNA was extracted with Trizol reagent (Vazyme, Nanjing, Jiangsu, China), which was reverse transcribed into cDNA to the manufacturer's instructions (Vazyme, Nanjing, Jiangsu, China). A SYBR Primer‐Script RT‒PCR Kit (Vazyme, Nanjing, Jiangsu, China) was used to examine the mRNA expression levels of the corresponding genes. β‐actin was used as an internal control to normalize the mRNA level of each gene. The primer sequences are listed in Table S2 (Supporting Information).

### Cell Proliferation and Colony Formation Assay

Four thousand cells were inoculated in 96‐wells plates, and cell proliferation was measured at different time points. Cells viability was determined with a Cell Counting Kit‐8 (Vazyme, Nanjing, Jiangsu China) following the manufacturer's instructions.

Transfected PC cells (1200 per well) were inoculated into 6‐well plates for further culture for 10 days. The samples were fixed with 4% PFA for 20 min after being washed twice with PBS, washed twice with PBS again and stained with 0.1% crystal violet for 20 min. Finally, the samples were rinsed with running water, and pictures were taken.^[^ ^46^ ^]^

### Transwell Assay

PANC‐1 and AsPC‐1 cells (5 × 10^4^ cells) were placed in the lower chamber of the Transwell system. The lower chamber was filled with DMEM supplemented with 20% FBS. After 48 h of culture, the cells in the upper chamber were washed with PBS. The cells were then fixed with 4% PFA for 20 min, and the fixed cells were stained with 0.1% crystal violet for 20 min. The results were then microscopically imaged.

### ECAR

The treated cells were evenly spread on a 96‐well XF cell culture plate (Agilent Technologies, Inc., CA) at a density of 5 × 10^4^ cells mL^−1^, with 80 µL of cell suspension per well and 4 or 8 wells per group. After 48 h of cultivation, the culture medium was discarded, and fresh assay medium containing different detection reagents was added to each well. The cartridge was loaded with glucose (10 mm), oligomycin (1 µm), and 2‐deoxyglucose (2‐DG, 50 mm) to determine the ECAR via a Seahorse XFe96 Cell Energy Metabolism Analyzer (Agilent Technologies, Inc., CA) at the specified time points.^[^ ^47^ ^]^

Vector Construction and Lentivirus‐Based Gene Transduction

For gene expression, the CDSs of HACD2, PKM2, and PRKN were amplified by reverse‐transcription PCR and then inserted into the pcDNA4/HisMax B, p3xFLAG‐CMV‐10, and eGFP‐C3 vectors or the pLenti9B vector with the indicated restriction enzymes. For gene silencing, shRNAs were annealed and ligated to Plvx‐Puro according to the manufacturer's instructions. The shRNA sequences used are listed in Table S3 (Supporting Information).

### Oil Red O Staining Assay

Cells (10 000 per well) were inoculated into 24‐well plates for further culture for 2 days. And then Oil Red O staining was performed with Oil Red O Kit following the manufacture's instruction.^[^ ^48^ ^]^

Mass spectrometry analysis of HACD2‐interacting proteins: HEK‐293T cells transfected with His‐HACD2 were subjected to IP with an anti‐His antibody or lgG, and then incubated with protein A/G (MedChemExpress, Shanghai, China) for 2 h at room temperature. After washing three times, the samples were denatured with 1 × loading buffer and resolved by SDS‒PAGE. The final mass spectrometry experiments and analysis of the results were performed by Biomarker Technol OG/ES (Beijing, China).

### ITC

The His‐tagged human HACD2 CDS region was inserted into the pFastBac1 vector and expressed in the *E. coli* strain (DH10Bac). The recombinant plasmid was extracted from the positive colonies and transferred into sf9 cells. The 96‐h culture supernatant was collected to infect the insect cells, and the target protein was harvested 48 h later. Ni‐NTA affinity chromatography was subsequently used for protein purification. The His‐HACD2 fusion protein was solubilized in 10 mm PBS. The sample cell was filled with 280 µL of human recombinant HACD2 protein at 10 mm, and the syringe was filled with 60 µL of orlistat (100 mm). The titration consisted of an initial injection of 4 µL followed by 13 injections of 3 µL. The data were analyzed by MicroCal PEAQ‐ITC analysis software.

### Ethic Approval and Consent to Participate

The study was conducted in accordance with the principles of the Declaration of Helsinki principles. It was approved by the Animal Use and Care Committees at China pharmaceutical University.

### Statistical Analysis

The results are shown as the means ± SD. All the statistical analyses were performed via GraphPad Prism 8. Paired two‐tailed Student's *t*‐tests, unpaired two‐tailed Student's *t*‐tests, one‐way ANOVA, and two‐way ANOVA test were used to analyze the differences among different groups. All the gel images of the relative protein expression were analyzed by ImageJ. Significant differences are indicated by *p < 0.05, **p < 0.01, and ***p < 0.001. Statistical details are included in the figure legends.

Public Data Analysis: GSE183795, GSE32059, GSE17576, GSE231509, GSE11398, GSE7338, GSE11979, GSE62452, GSE28735, and GSE211398 were obtained from GEO datasets of the National Center for Biotechnology Information (https://www.ncbi.nlm.nih.gov/). The differential expression levels of HACD1‐4, SPARC, MGLL COL4A1, SERPINH1, PLBD1, and ACADL in tumor and normal tissues were analyzed via GEPIA, and the correlations between HACD2 and PKM2/LDH/PFKFB3/GLUT1, and the correlations between gene expression and prognosis were also analyzed via GEPIA^[^ ^49^ ^]^ (http://gepia.cancer‐pku.cn/). Differential expression levels of PRKN in pancreatic tissues versus normal tissues were obtained from the UCLAN database^[^ ^50^, ^51^ ^]^ (https://ualcan.path.uab.edu/). The relationship between PRKN expression and prognosis was analyzed via Kaplan–Meier plotter^[^ ^52^ ^]^ (https://kmplot.com/analysis/). The orlistat structural formula was drawn via ChemDraw software, and some pictures in the mechanism pathway diagram were drawn by Figdraw.

## Conflict of Interest

The authors declare no conflict of interest.

## Author Contributions

Y.Z. and L.M. designed the experiments. X.C. performed the in vitro experiments and wrote the manuscript. J.Z. helped in vivo research. Y.S. and Q.F. analyzed data. C.Z. supervised the project.

## Supporting information



Supporting Information

## Data Availability

The data that support the findings of this study are available from the corresponding author upon reasonable request.
