# Supplementary material for: HACD2 Promotes Pancreatic Cancer Progression by Enhancing PKM2 Dissociation From PRKN in a Dehydratase‐Independent Manner
Source: Adv Sci (Weinh). 2025 Jan 21;12(10):2407942. doi: 10.1002/advs.202407942 (PMC11904967; doi:10.1002/advs.202407942)
Supplement: Supplementary file 1 — Supporting Information [file ADVS-12-2407942-s001.pdf]

ADVANCED SCIENCE
Open Access

Supporting Information

for *Adv. Sci.*, DOI 10.1002/advs.202407942

HACD2 Promotes Pancreatic Cancer Progression by Enhancing PKM2 Dissociation From PRKN in a Dehydratase-Independent Manner

Xuanning Chu, Jinyu Zhao, Yuting Shen, Qi Feng, Changlin Zhou, Lingman Ma* and Yiran Zhou**

Figure S1. Bioinformatic analysis of potential target genes involved in PC progression. A,B) Differential expression and survival analysis of genes in the GEPIA database. C,D) The GEPIA dataset was used to assess differential expression and perform survival analysis of HACD2 in other tumors. E,F) The GEPIA dataset revealed the expression of other HACD2 subtypes and survival analysis in patients with PC.

Figure S2. HACD2 silencing and overexpression efficacy. A,B) The knockdown efficacy in AsPC-1 and PANC-1 cells was verified via RT–qPCR (n = 4) and western blotting. Data were presented as means ± SD. *, $p < 0.05$, **, $p < 0.01$, ***, $p < 0.001$ according to one-way ANOVA. C,D) The overexpression efficacy was verified by RT–qPCR (n = 4) and western blotting in PANC-1 cells. Data were presented as means ± SD. * $p < 0.05$, ** $p < 0.01$, *** $p < 0.001$ according to unpaired two-tailed Student' *t* test. E,F) Transwell and flow cytometric assays were used to detect cell migration and apoptosis after HACD2 knockdown. Scale bar, 100 μm.

Figure S3. HACD2 is correlated with lipid synthesis in PC**.** A) Venn diagram showing the strategy for identifying target genes associated with DEGs in PC patients with $p < 0.05$ in each dataset. (https://www.ncbi.nlm.nih.gov/). B) GO analysis of DEGs. C) LC-MS quantification of VLCFAs in PANC-1 cells (vector control or HACD2 knockdown). n = 4. D) The relative TG content was analyzed in AsPC-1 and PANC-1 cells (vector control or HACD2 knockdown). The cells were analyzed 48 hours after adherence. Data were presented as means ± SD. * $p < 0.05$, ** $p < 0.01$, *** $p < 0.001$ according to unpaired two-tailed Student's *t* test. n = 3. E) The relative TG content was analyzed in control, $HACD2^{WT}$ or $HACD2^{MUT}$ AsPC-1 and PANC-1 vector cells. The cells were analyzed 48 hours after adherence. The cells were analyzed after 48 hours of adherence. n = 3. Data

were presented as means ± SD. ns., not significant.

Figure S4. HACD2 enhances PKM2/c-Myc-mediated glycolysis in PC. A,B) The relative glucose intake and lactate release by PC cells with HACD2 knockdown or overexpression were monitored. n = 3. Data were presented as means ± SD. * $p < 0.05$, ** $p < 0.01$, *** $p < 0.001$ according to unpaired two-tailed Student's *t* test. C) GO analysis of the HACD2 interactome. (https://metascape.org/). D) Correlation analysis between HACD2 and the mRNA expression of other glycolytic genes based on the GEPIA database. (http://gepia2.cancer-pku.cn). E) Glycolysis-related mRNA levels were assessed by RT–qPCR in PC cells after HACD2 knockdown. n = 4. Data were presented as means ± SD. ns., not significant; * $p < 0.05$, *** $p < 0.001$ according to unpaired two-tailed Student's *t* test.

Figure S5. HACD2 knockdown inhibits PKM2 dimerization. A) Western blotting was used to determine the monomer and dimerization levels of PKM2 after CQ and MG132 treatment following HACD2 knockdown. B) Western blotting was used to determine the protein expression levels of PKM2 in the cytoplasm and nucleus after MG132 and CQ were added to HACD2-knockdown cells. C) IF was used to assess the nuclear translocation of PKM2 in HACD2-knockdown PANC-1 and AsPC-1 cells after CQ and MG132 treatment. Scale bar, 20 μm. D) The protein expression levels of PKM2 dimerization and c-Myc after PKM2 overexpression and HACD2-knockdown, as shown by western blotting.

Figure S6. HACD2 regulates PKM2 dimerization and ubiquitination in a fatty acid dehydratase-independent manner. A) The mRNA expression levels of HACD2, PKM2 dimer and c-Myc were assessed via RT–qPCR after HACD2-knockdown cells were treated with 2 μM VLCFAs for 24

hours. n = 4. Data were presented as means ± SD. ns., not significant. B) Immunoprecipitation was used to assess the effect of 2 μM VLCFAs on PKM2 ubiquitination in HACD2-knockdown cells. C) Degradation of the PKM2 protein in HACD2^{WT} and HACD2^{MUT}-overexpression PC cells was measured via CHX chase analysis. n = 3. Data were presented as means ± SD. *** $p < 0.001$ according to two-way ANOVA. D) The protein expression levels of HACD2, the PKM2 dimer and c-Myc were assessed via western blotting after HACD2-knockdown cells were treated with 2 μM VLCFAs for 48 hours. E) PKM2 monomers and dimers in AsPC-1 and PANC-1 cells transduced with HACD2^{WT}, HACD2^{MUT}, or a vector control were detected by western blotting. F) Western blotting was used to assess the overexpression efficacy of HACD2 and the knockdown efficacy of PKM2 in PANC-1 cells.

Figure S7. HACD2 directly binds to PRKN and enhances the dissociation of PKM2 from PRKN. A) Coomassie brilliant blue staining of His-HACD2 pull-down material from HEK-293T cells and a Venn diagram showing PKM2 binding partners determined by HitPridict and BioGIRD and HACD2-binding protein interactions determined by IP-MS. (https://www.hitpredict.org/, https://thebiogrid.org/). B) PRKN was immunoprecipitated from PANC-1 cells transduced with shHACD2 or a control vector and analyzed via western blotting to assess the binding of endogenous PKM2 and HACD2 to PRKN. C) Immunoprecipitation was used to detect the binding of PKM2 and HACD2 to PRKN in the context of simultaneous overexpression of HACD2 and PRKN. D) His-HACD2, GFP-PRKN and Flag-PKM2 were immunoprecipitated dose-dependently from HEK-293T cells via an anti-GFP antibody and analyzed by western blotting. E) Immunoprecipitation was used to assess the effect of PRKN on HACD2 ubiquitination.

Figure S8. PRKN is expressed at low levels in PC. A) Immunohistochemistry staining of PRKN in paired paracarcinoma and primary tumors from PC patients. B) HACD2 expression in different PC cell lines was verified by western blotting. C) Western blotting was used to assess the overexpression efficacy of PRKN and HACD2 in PANC-1 cells.

Figure S9. Orlistat acts on sites 165 and 242 of HACD2. A) Western blotting was used to determine the protein expression levels of HACD2, PRKN and PKM2 in response to increasing doses of orlistat. B) MOE was used to predict the binding site of orlistat to HACD2. C) The relative TG content was analyzed in PANC-1 cells (vector control, HACD2 knockdown, $HACD2^{WT}$, $HACD2^{165MUT}$, $HACD2^{242MUT}$ and $HACD2^{165/242MUT}$). The cells were analyzed 48 hours after adherence. n = 3. Data were presented as means ± SD. * $p < 0.05$, ** $p < 0.01$, *** $p < 0.001$ according to one-way ANOVA. D) The Oil Red O staining was analyzed in PANC-1 cells (vector control, HACD2 knockdown, $HACD2^{WT}$, $HACD2^{165MUT}$, $HACD2^{242MUT}$ and $HACD2^{165/242MUT}$). The cells were analyzed 48 hours after adherence. Scale bar, 20 μm. E) Images of orlistat-treated PANC-1 cells after HACD2 mutation (vector control, HACD2 knockdown, $HACD2^{WT}$, $HACD2^{165MUT}$, $HACD2^{242MUT}$ and $HACD2^{165/242MUT}$). Scale bar, 100 μm. F) CCK-8 assays were used to determine the cell viability and proliferation on orlistat treated after HACD2 mutation (vector control, HACD2 knockdown, $HACD2^{WT}$, $HACD2^{165MUT}$, $HACD2^{242MUT}$ and $HACD2^{165/242MUT}$) of PANC-1 cells. n = 4. Data were presented as means ± SD. ns., not significant; * $p < 0.05$, ** $p < 0.01$, *** $p < 0.001$ according to one-way ANOVA. G) The ubiquitination of PKM2 in HACD2-mutant PANC-1 cells was measured by immunoprecipitation after orlistat treatment.

Figure S1. Bioinformatic analysis of potential target genes involves in PC progression

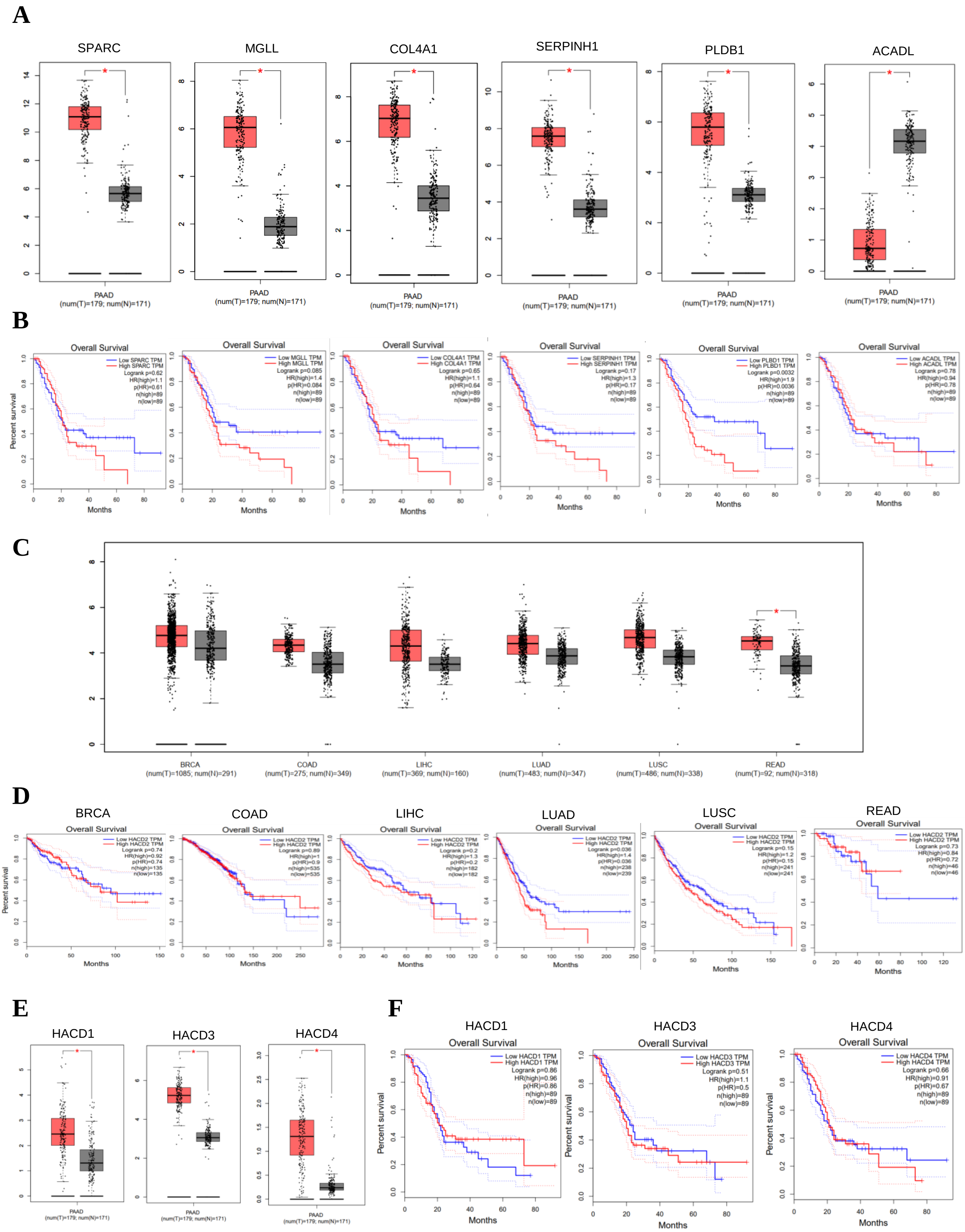

Figure S2. HACD2 silencing and overexpression efficacy

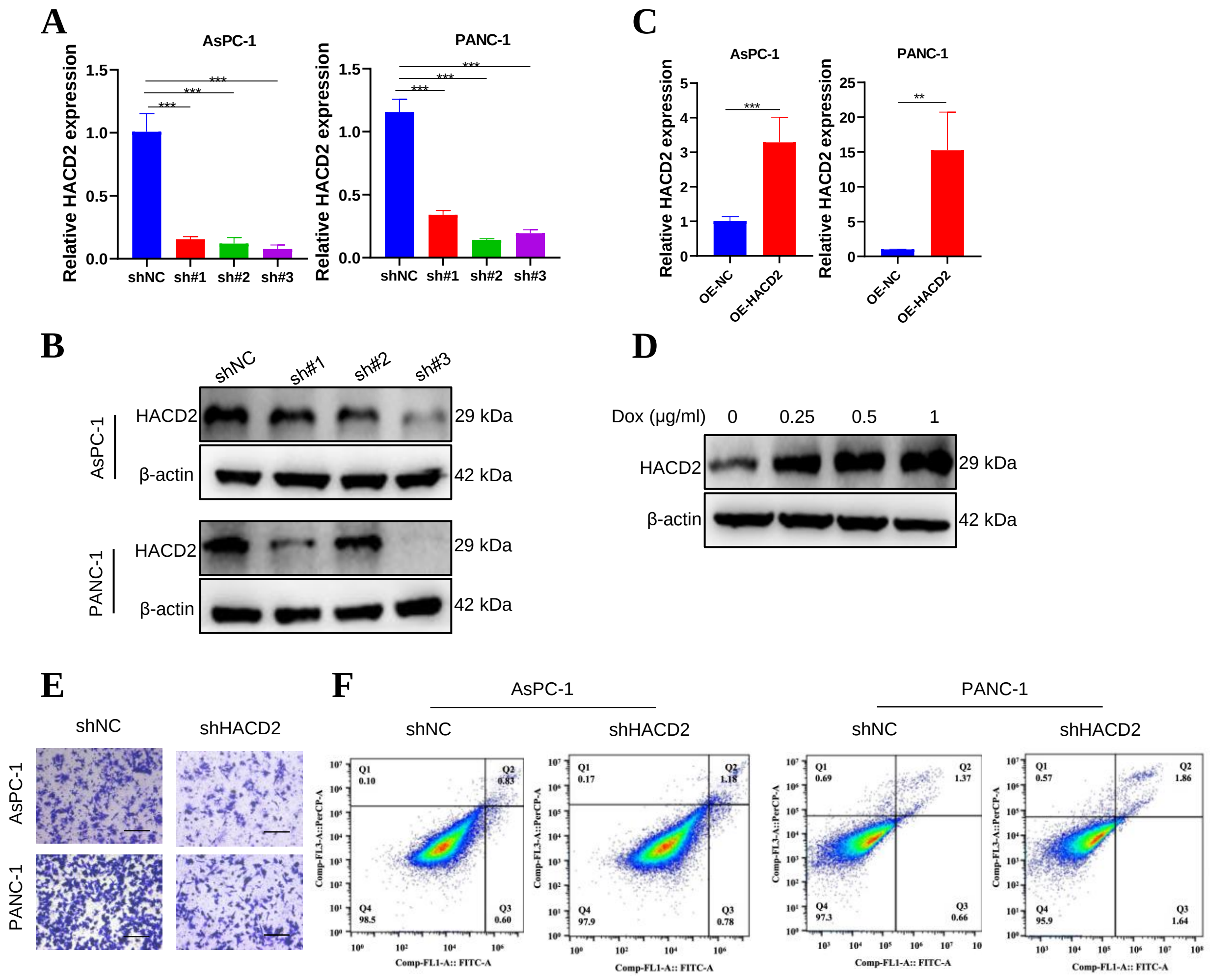

Figure S3. HACD2 is correlated with lipid synthesis in PC

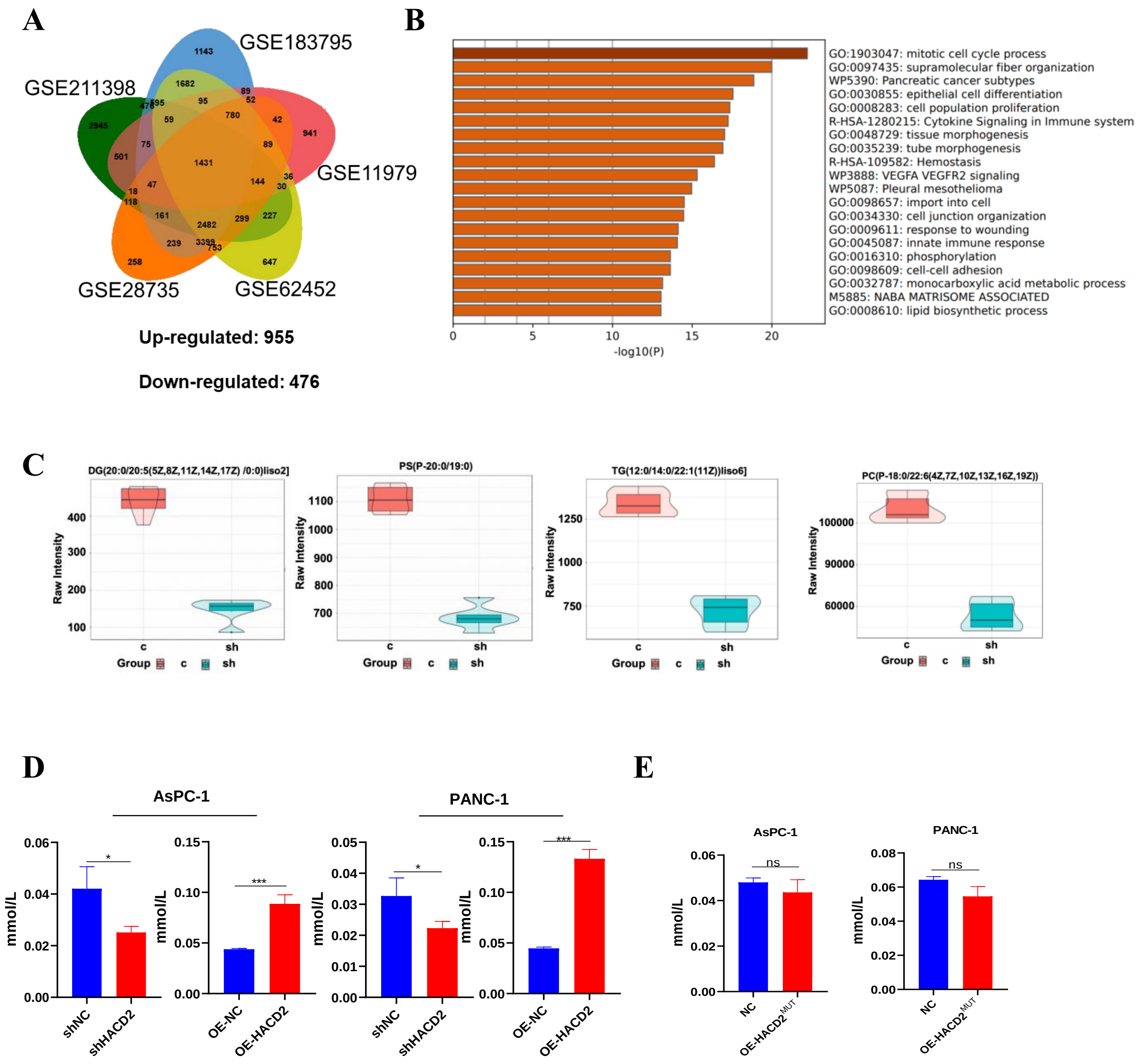

Figure S4. HACD2 enhances PKM2/c-Myc-mediated glycolysis in PC

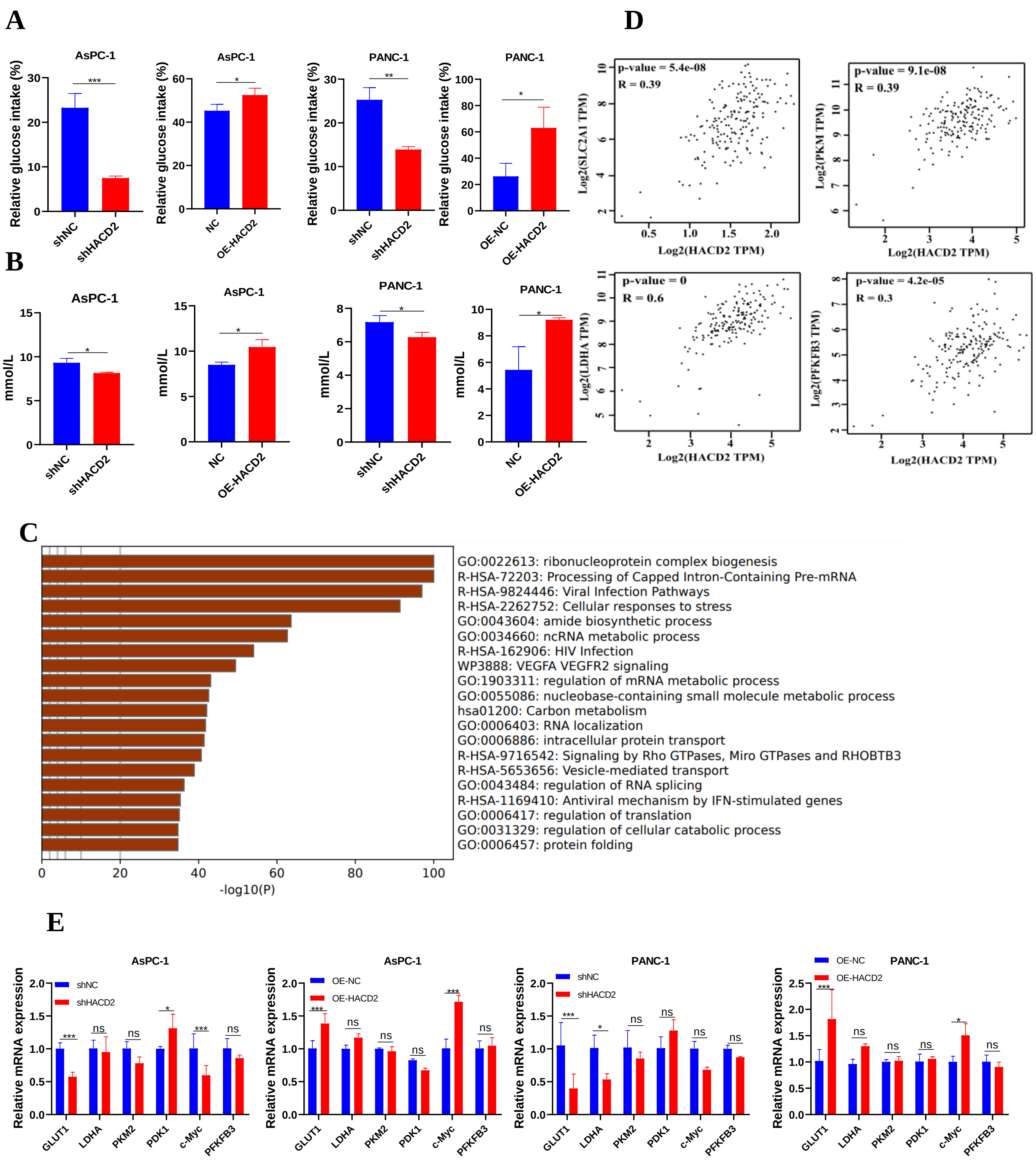

Figure S5. HACD2 knockdown inhibits PKM2 dimerization

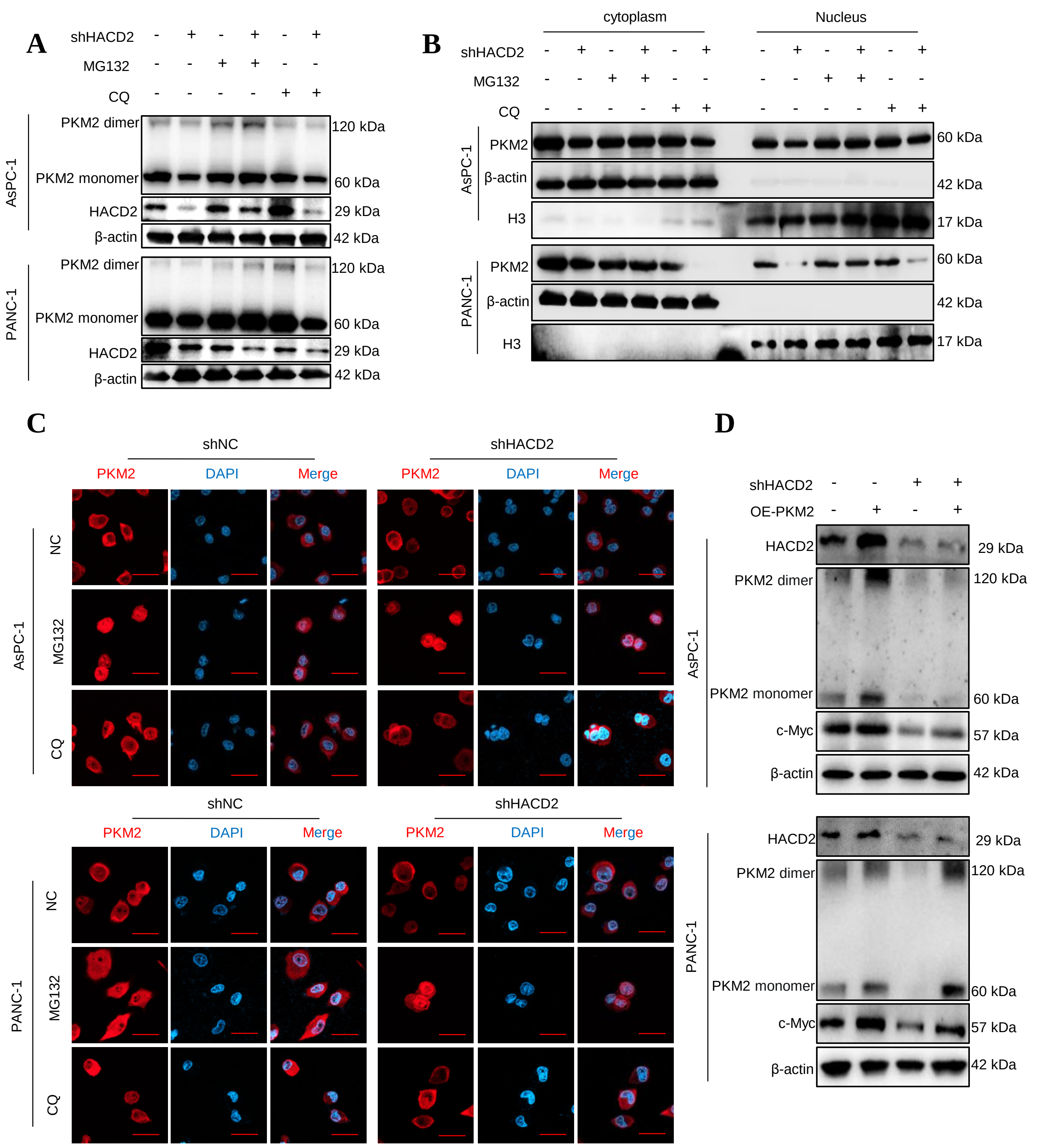

Figure S6. HACD2 regulates PKM2 dimerization and ubiquitination in a fatty acid dehydratase-independent manner

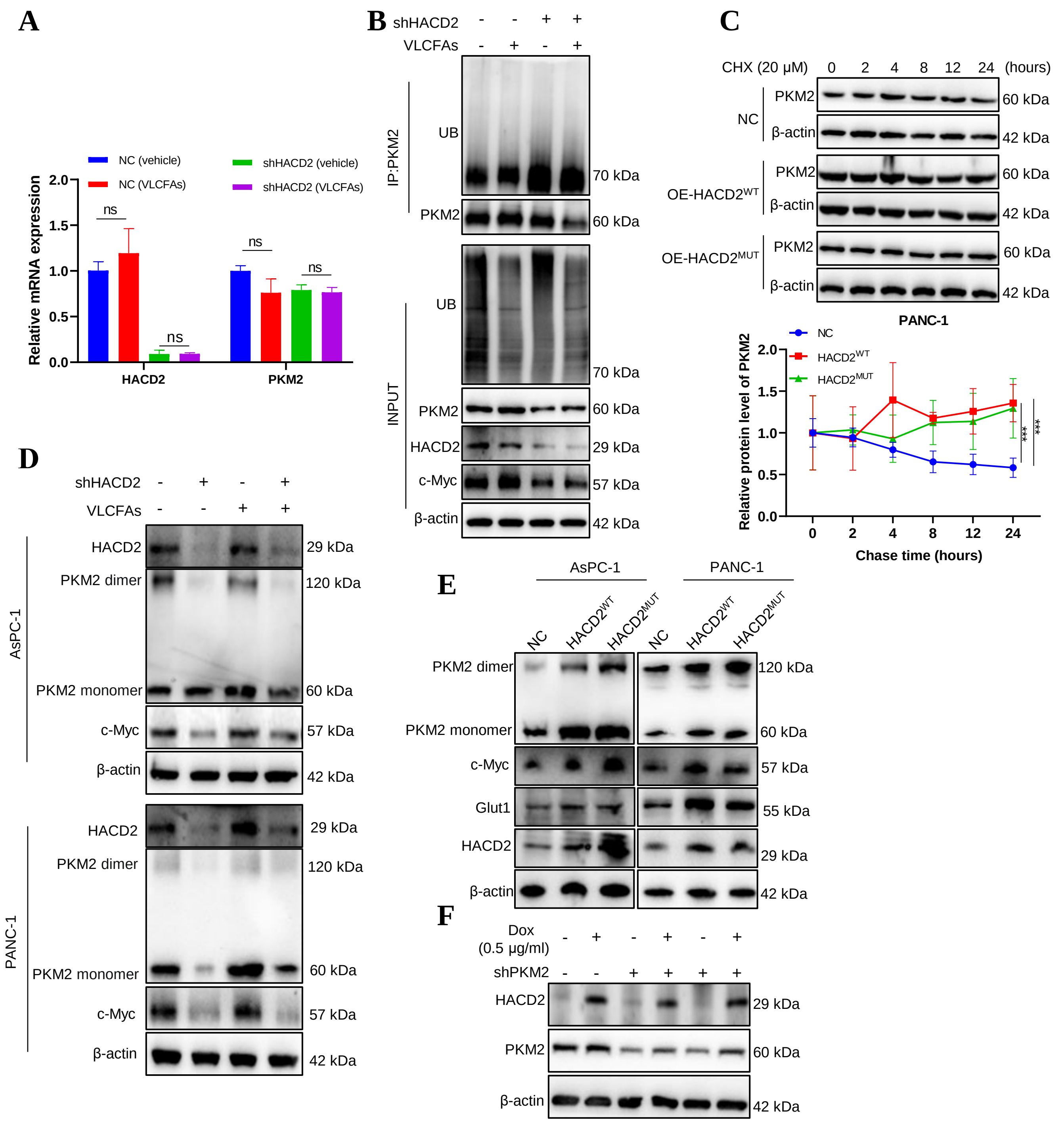

Figure S7. HACD2 directly binds to PRKN and enhances the dissociation of PKM2 from PRKN

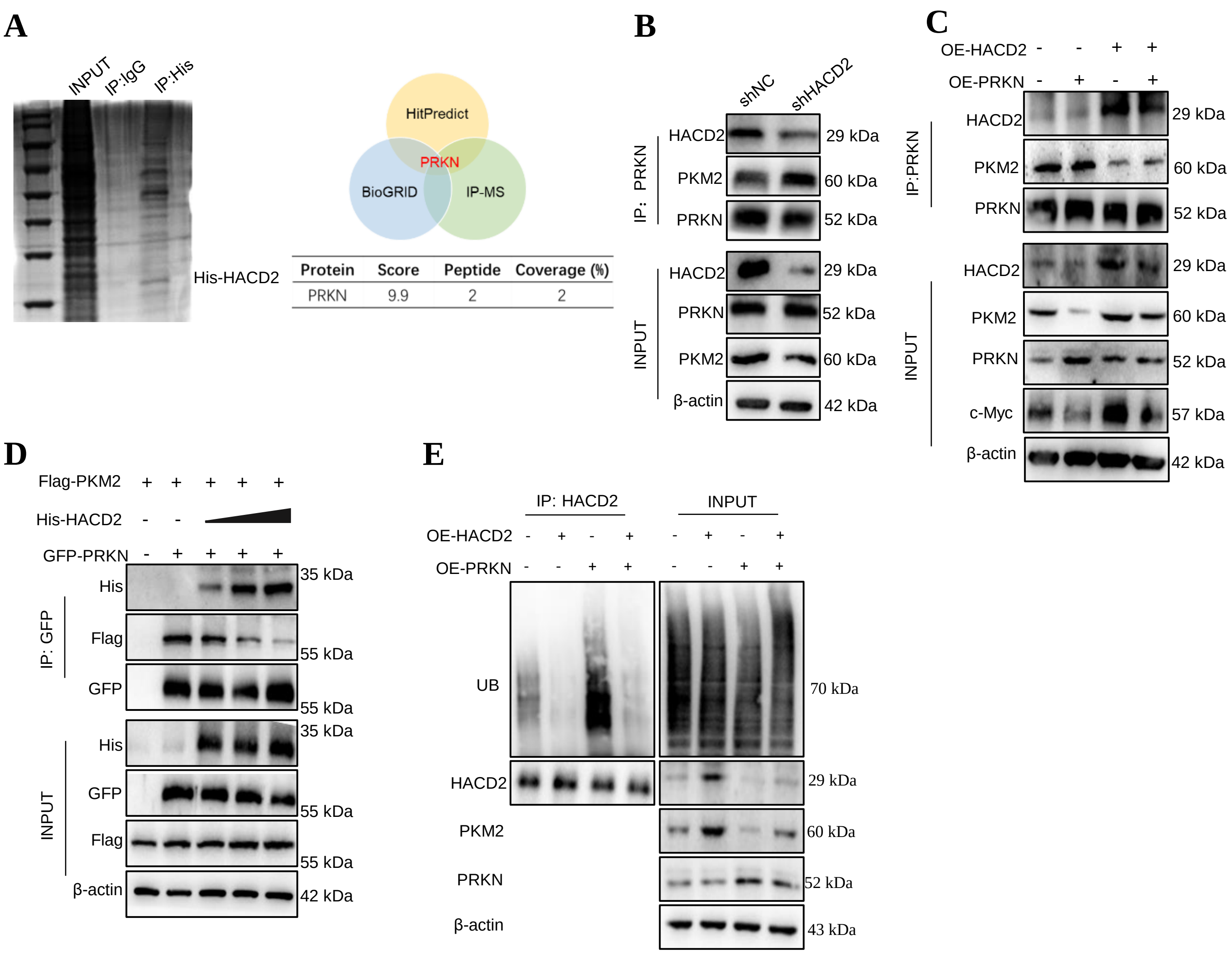

Figure S8. PRKN is expressed at low levels in PC

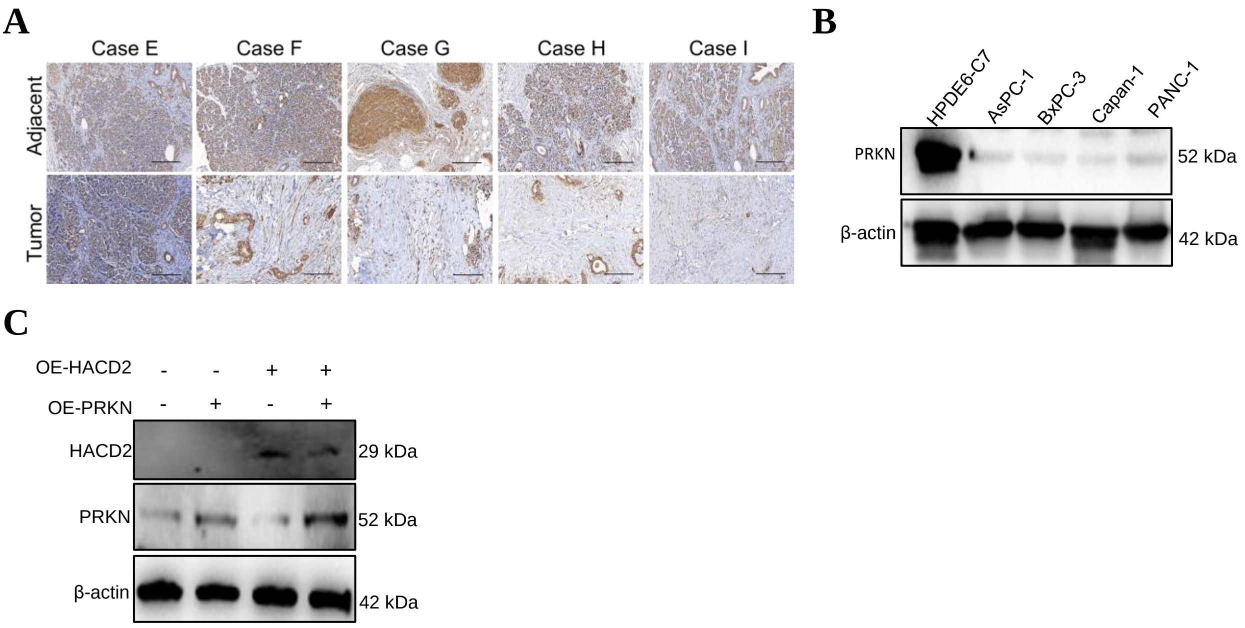

Figure S9. Orlistat acts on sites 165 and 242 of HACD2

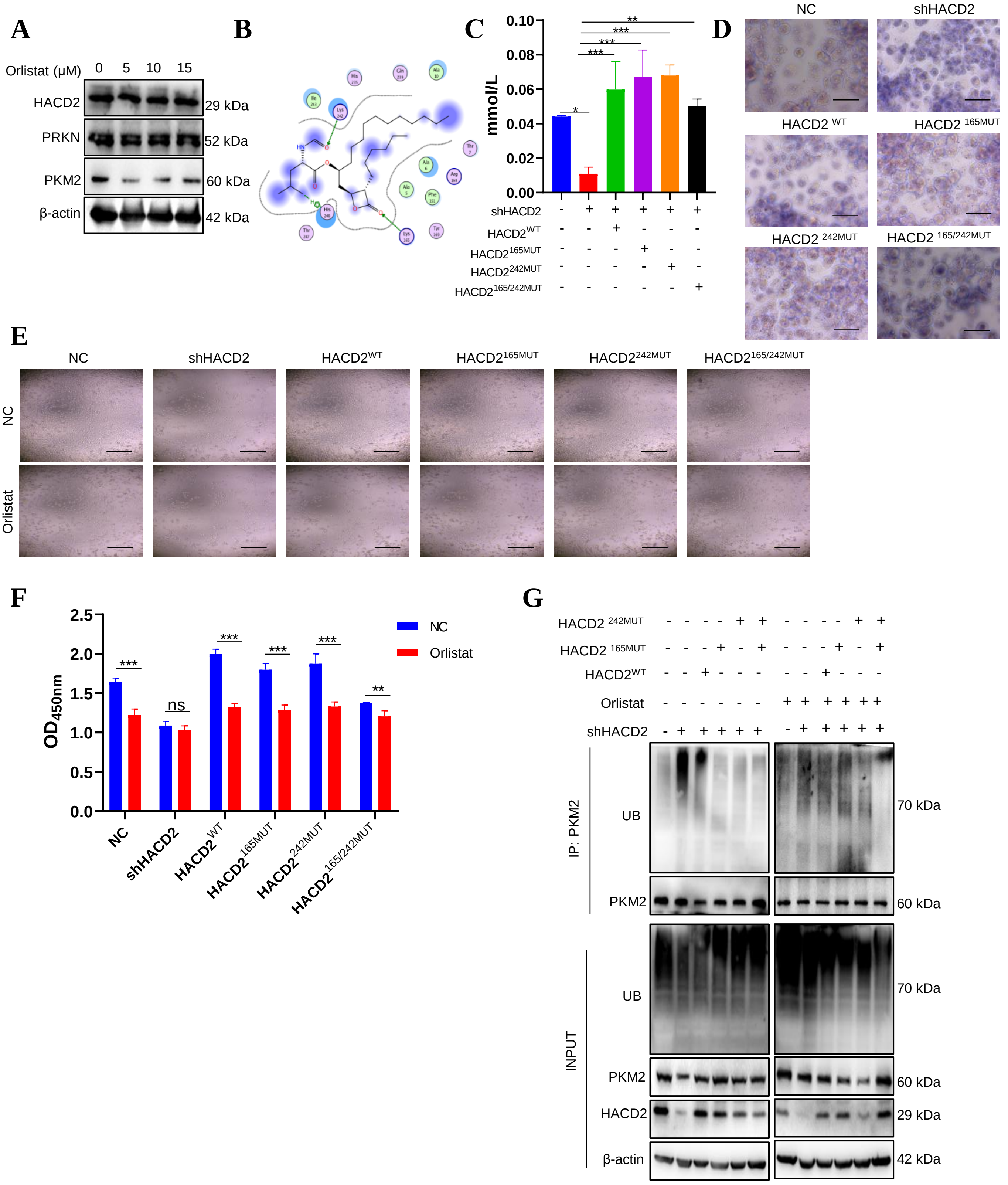

Table S1. Statistical table of 83 pancreatic cancer patients by HACD2 staining intensity.

Characteristics	Number of Patients		Odds ratio	P-value
	High HACD2 Expression	Low HACD2 Expression		
Age			1.400	0.4454
< 60	21	18		
⩾60	20	24		
Gender			0.7867	0.5856
Female	21	23		
Male	20	19		
Tumor stage				0.0001
I	8	12		
II	31	13		
III	2	9		
IV	0	8		
Survival (Month)			2.541	0.0373
⩽13	24	15		
> 13	17	27		

Significance was determined by a chi-squared test.

Table S2. Primers used in this study.

Gene	Forward primer (5'-3')	Reverse primer (5'-3')
β-actin	CATGTACGTTGCTATCCAGGC	CTCCTTAATGTCACGCACGAT
HACD2	GCAGTAACACATAGCGTCAAAGA	TGATGAGGTAAGGCAGATGGTT
PKM2	ATGTCGAAGCCCCATAGTGAA	TGGGTGGTGAATCAATGTCCA
c-Myc	GTCAAGAGGCGAACACACAAC	TTGGACGGACAGGATGTATGC
Glut1	ATTGGCTCCGGTATCGTCAAC	GCTCAGATAGGACATCCAGGGTA
LDHA	ATGGCAACTCTAAAGGATCAGC	CCAACCCCAACAACTGTAATCT
PFKFB3	ATTGCGGTTTTCGATGCCAC	GCCACAACTGTAGGGTCGT

Table S3. shRNA sequences used in this study.

Gene	Sequences
shHACD2-1	Forward: CCGGCCAGTTATACTTCCACATGATCTCGAGATCATGTGGAAGTATAACTGGTTTTTG Reverse: AATTCAAAAACCAGTTATACTTCCACATGATCTCGAGATCATGTGGAAGTATAACTGG
shHACD2-2	Forward: CCGGCTATAGGAATTGTTCCATCTTCTCGAGAAGATGGAACAATTCCTATAGTTTTTG Reverse: AATTCAAAAACTATAGGAATTGTTCCATCTTCTCGAGAAGATGGAACAATTCCTATAG
shHACD2-3	Forward: CCGGCAGACAAGCTGGCCTATATTCCTCGAGGAATATAGGCCAGCTTGTCTGTTTTTG Reverse: AATTCAAAAACAGACAAGCTGGCCTATATTCCTCGAGGAATATAGGCCAGCTTGTCTG
shPKM2-1	Forward: CCGCGCCCGAGGCTTCTTCAAGAAGCTCGAGCTTCTTGAAGAAGCCTCGGGCTTTTTG Reverse: AATTCAAAAAGCCCGAGGCTTCTTCAAGAAGCTCGAGCTTCTTGAAGAAGCCTCGGGC
shPKM2-2	Forward: CCGCGTTCGGAGGTTTGATGAAATCCTCGAGGATTTCATCAAACCTCCGAACTTTTTG Reverse: AATTCAAAAAGTTCGGAGGTTTGATGAAATCCTCGAGGATTTCATCAAACCTCCGAAC
